# A Cold-Inducible DEAD-Box RNA Helicase from *Arabidopsis thaliana* Regulates Plant Growth and Development under Low Temperature

**DOI:** 10.1371/journal.pone.0154040

**Published:** 2016-04-26

**Authors:** Yuelin Liu, Daisuke Tabata, Ryozo Imai

**Affiliations:** 1 Hokkaido Agriculture Research Center, National Agriculture and Food Research Organization, Sapporo, Japan; 2 Graduate School of Agriculture, Hokkaido University, Sapporo, Japan; Iwate University, JAPAN

## Abstract

DEAD-box RNA helicases comprise a large family and are involved in a range of RNA processing events. Here, we identified one of the *Arabidopsis thaliana* DEAD-box RNA helicases, AtRH7, as an interactor of *Arabidopsis* COLD SHOCK DOMAIN PROTEIN 3 (AtCSP3), which is an RNA chaperone involved in cold adaptation. Promoter:*GUS* transgenic plants revealed that *AtRH7* is expressed ubiquitously and that its levels of the expression are higher in rapidly growing tissues. Knockout mutant lines displayed several morphological alterations such as disturbed vein pattern, pointed first true leaves, and short roots, which resemble ribosome-related mutants of *Arabidopsis*. In addition, aberrant floral development was also observed in *rh7* mutants. When the mutants were germinated at low temperature (12°C), both radicle and first leaf emergence were severely delayed; after exposure of seedlings to a long period of cold, the mutants developed aberrant, fewer, and smaller leaves. RNA blots and circular RT-PCR revealed that 35S and 18S rRNA precursors accumulated to higher levels in the mutants than in WT under both normal and cold conditions, suggesting the mutants are partially impaired in pre-rRNA processing. Taken together, the results suggest that AtRH7 affects rRNA biogenesis and plays an important role in plant growth under cold.

## Introduction

In eukaryotes, ribosome biosynthesis is a complicated cellular process that sequentially takes place in the nucleolus, nucleoplasm, and cytoplasm [1,2]. Ribosome biogenesis includes transcription of ribosomal DNA to pre-rRNA, followed by rRNA processing and modification, and the assembly of rRNA with ribosomal and non-ribosomal proteins to form preribosomes, which then mature in the cytoplasm [2,3]. A large number of *trans*-acting factors, including H/ACA-box snoRNPs, C/D-box snoRNPs, endo- and exo-RNases, RNA helicase, kinases, AAA-type ATPase, ABC proteins and GTPases, are involved in these processes [2,4,5].

DEAD-box proteins comprise the largest family of RNA helicases, and exist in most organisms [6,7]. They possess 12 conserved motifs that are involved in ATPase, helicase, and RNA-binding activities, and participate in a variety of RNA-associated events from transcription to RNA decay [8,9]. To date, many DEAD box proteins have been shown to be involved in ribosome biogenesis. Five DEAD-box proteins exist in *Escherichia coli*, and four of them are involved in ribosome biogenesis in different ways [10,11]. For instance, deletion of *csdA* results in a decrease in the 50S subunit and an increase in the 40S subunit, as well as suppression of growth under low temperature [12]. In *Saccharomyces cerevisiae* (yeast), 15 DEAD-box proteins are required for different steps in ribosome biogenesis [6]. In contrast to *E*.*coli*, DEAD-box protein functions in ribosome biogenesis are essential for yeast survival under normal growth conditions [6]. The *Homo sapiens* (human) DEAD-box family contains 36 members, and several of them are essential for ribosome biogenesis [13].

Plants have a large family of DEAD-box proteins; the *Arabidopsis* genome encodes 58 DEAD-box proteins [14]. A number of *Arabidopsis* DEAD box RNA helicases play important roles in plant abiotic and biotic stress tolerance via their functions in specific RNA processing events [15–20]. Some DEAD-box proteins are involved in the regulation of plant growth and development through ribosome biogenesis [21–27]. For example, AtRH36/SWA3 is involved in 18S rRNA processing and controls female gametogenesis [25,26]. AtRH57 affects small ribosomal subunit formation and rRNA processing, and its mutants show enhanced sensitivity to glucose and ABA [27]. Three DEAD-box proteins, AtRH39, AtRH3 and AtRH22, are specifically involved in chloroplast rRNA biogenesis [21–24]. Despite recent extensive studies on DEAD-box proteins, the functions of many members remain uninvestigated.

Plant cold shock domain (CSD) proteins are RNA chaperones that destabilize RNA secondary structures [28]. One of the *Arabidopsis* CSD proteins, AtCSP3, is induced during cold acclimation and serves as an RNA chaperone *in vivo* [29]. A loss-of-function mutant of AtCSP3 (*atcsp3-2*) is sensitive to freezing, while *AtCSP3* overexpressors display enhanced tolerance against freezing [29]. Therefore, AtCSP3 is considered to be a positive regulator of freezing tolerance [29]. AtCSP3 interacts with several different nuclear and cytoplasmic proteins that are involved in RNA metabolism, suggesting that AtCSP3 participates in a wide range of RNA processing events within the cells [30].

Here, we analyzed biological functions of AtRH7, an interactor of AtCSP3 [30]. Knockout mutants of *AtRH7* displayed several morphological alterations during vegetative and reproductive growth. In addition, the mutants exhibited severe defects in germination and leaf development under long-term low temperature conditions. Accumulation of rRNA precursors in *rh7* mutant plants corroborated the hypothesis that AtRH7 affects ribosome biogenesis.

## Results

### Identification of AtRH7 as an interactor of AtCSP3

**Fig 1:**
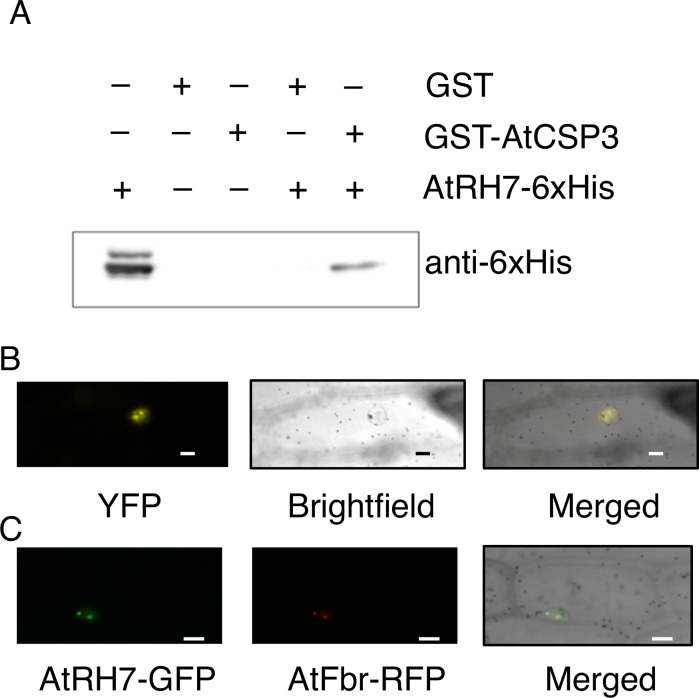
AtRH7 is an interactor of AtCSP3. (A) *In vitro* pull-down assay of AtRH7-6xHis protein with GST or GST-AtCSP3 fusion proteins. GST-AtCSP3 and GST proteins were used as bait to pull down the AtRH7-6xHis protein from the induced *E*.*coli* extracts. The immunoblot detection of prey protein was performed using anti-6xHis. Aliquots of AtRH7-6xHis input (10%) is shown. (B) BiFC assay analysis of the interaction between AtRH7 and AtCSP3 *in vivo*. The plasmids pSAT4-nEYFP-AtCSP3 and pSAT4-cYFP-AtRH7 were bombarded into onion epidermal cells, and the cells were observed after a 16-h incubation at 22°C in dark. (C) Co-subcellular localization of AtRH7-GFP and AtFbr-RFP (nucleolus marker). Scale bars = 20μm.

Using AtCSP3 as a bait in a yeast two-hybrid screen, we previously identified several potential interactors including the DEAD-BOX RNA helicase AtRH7/PRH75 (At5g62190) [30]. To verify the interaction between AtCSP3 and AtRH7, we first performed an *in vitro* pull-down assay. As shown in Fig 1A, AtRH7-6xHis was able to interact with GST-AtCSP3, but not with GST alone. We also utilized bi-molecular fluorescence complementation (BiFC) assays to confirm the interaction *in vivo*. Onion cells were co-bombarded with nYFP-AtCSP3, where AtCSP3 was fused to the N-terminus of YFP, and cYFP-AtRH7, where AtRH7 was fused to the C-terminus of YFP. Reconstituted YFP signals, indicating interaction between AtCSP3 and AtRH7, were observed within the nucleus of the transformed cells (Fig 1B). This YFP signal pattern was similar to the GFP signal from onion cells transformed with AtRH7-GFP (Fig 1C). By comparing signals from the nucleolus marker (AtFbr-RFP), it was concluded that AtRH7 localizes mainly to the nucleolus and to a minor degree to the nucleoplasm (Fig 1C). Together, these data indicated that AtRH7 forms a complex with AtCSP3 mainly in the nucleolus, with a smaller portion in the nucleoplasm.

### Phylogenetic analysis of AtRH7

**Fig 2:**
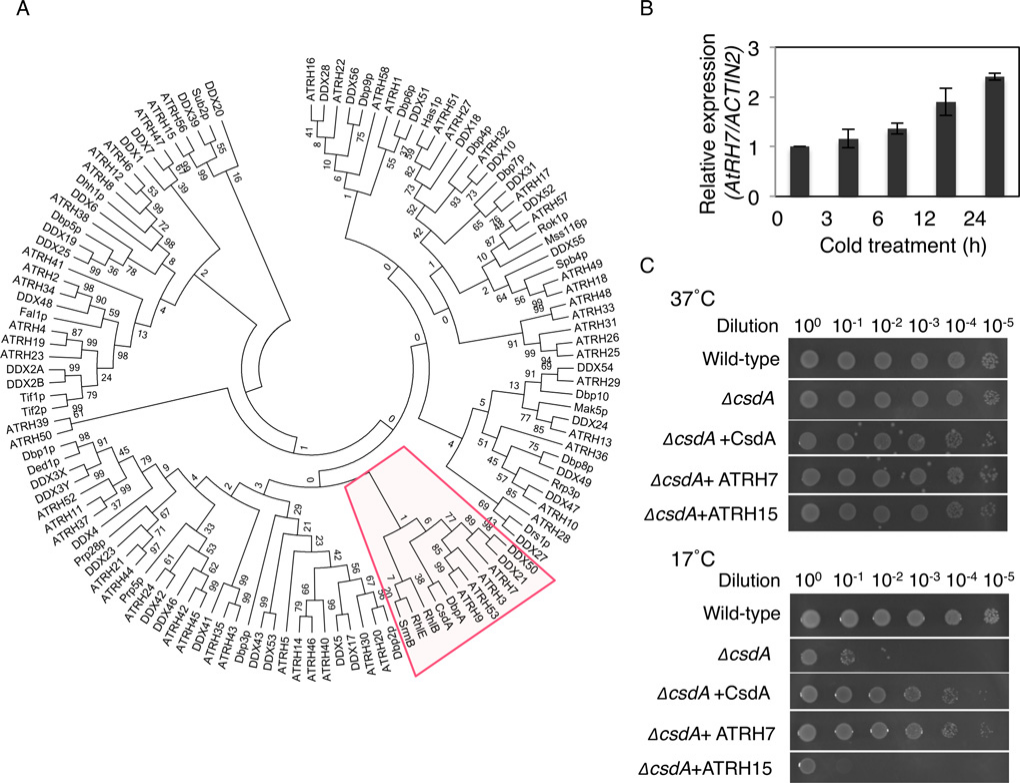
Complementation assay of AtRH7 in *E*.*coli ΔcsdA* mutant. (A) Phylogenetic tree of DEAD-box helicase family members from *Arabidopsis*, human, yeast and *E*. *coli;* only core helicase domains were analyzed. Red box indicates the clade that includes AtRH7. (B) Quantitative real-time PCR (qRT-PCR) analysis of expression of *AtRH7* in Col-0 wild-type seedlings with cold treatment. Expression of *AtRH7* was normalized to expression of *ACT2*. The data represent the means of three independent experiments ± SD. (C) Complementation ability of *AtRH7* in the cold-sensitive *E*.*coli ΔcsdA* mutant. The wild-type, *ΔcsdA* mutant and *ΔcsdA* mutant cells harboring pINIII-*CsdA* (positive control), pINIII-*AtRH7* and pINIII-*AtRH15* were spotted on LB-agar plates and the cells were incubated at 37°C and 17°C for 14 h and 72 h hours, respectively.

To address the evolutionary and functional relationships of AtRH7, a phylogenetic tree was created for DEAD-box RNA helicases from *Arabidopsis* [14,31], human [32], yeast [8] and *E*.*coli* [33], utilizing the core helicase regions of proteins without N- and C- terminal extension sequences (Fig 2A). AtRH7 formed a clade with three other *Arabidopsis*, three human and five *E*. *coli* DEAD-box RNA helicases. AtRH7 was most closely related to human DDX21 and DDX50, which participate in ribosome biogenesis [34]. Within *Arabidopsis*, AtRH7 was closely related to AtRH3, AtRH9/PMH1 and AtRH53/PMH2. AtRH3 is a chloroplast protein involved in group II intron splicing and ribosome biogenesis [21,22]. AtRH9 and AtRH53 are associated with a large complex in mitochondria [35], and AtRH53 is required for group II intron splicing in mitochondria [36]. It is interesting to note that all five *E*. *coli* DEAD-box RNA helicases are present in this clade, while this clade lacks yeast DEAD-box RNA helicases. Together, the phylogenetic analysis indicated that AtRH7 belongs to a family whose members are involved in rRNA and mRNA processing.

### AtRH7 complements the cold-sensitive phenotype of the *E*. *coli csdA* mutant

Given that we found AtRH7 to be an interactor of AtCSP3, we determined the expression of *AtRH7* in response to cold. qRT-PCR analysis revealed that the transcript levels of *AtRH7* gradually increased in response to cold and were induced 2.3-fold after 24 h of cold treatment (Fig 2B). Since one of the *E*. *coli* AtRH7 homologues, CsdA (cold shock DEAD-box protein A), is cold inducible and has a function associated with CSD proteins in *E*. *coli* [12,33], we tested whether there is functional conservation between CsdA and AtRH7. Accordingly, we performed a complementation assay using the *E*. *coli ΔcsdA* mutant. The *ΔcsdA* mutant is deficient in growth at low temperatures [33]. The mutant containing the vector alone exhibited suppressed growth at 17°C, whereas the growth suppression could be relieved by expression of *AtRH7* or *CsdA*. By contrast, AtRH15, another DEAD-box RNA helicase that interacts with AtCSP3 [30], could not complement the cold sensitivity of the *ΔcsdA* mutant (Fig 2C). This result suggested that AtRH7 shares a conserved function with *E*.*coli* CsdA under cold conditions.

### *AtRH7* is expressed ubiquitously in plants, with higher levels in rapidly developing tissues

**Fig 3:**
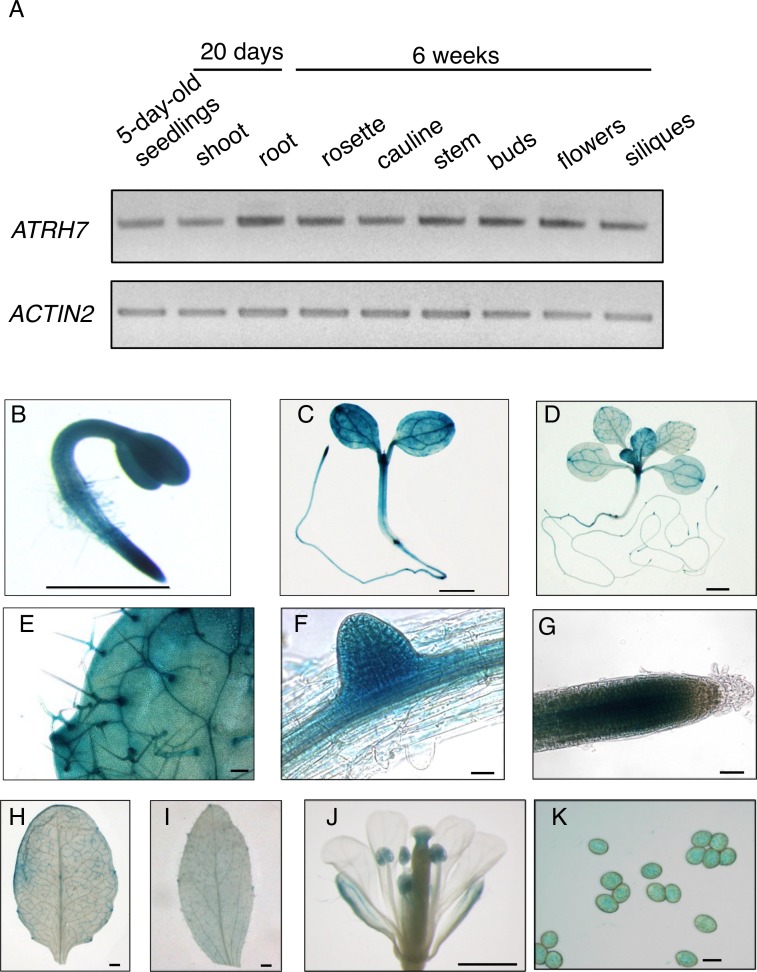
Expression pattern of *ATRH7* in different tissues. (A) Semi-quantitative RT-PCR analysis of *AtRH7* expression in different tissues. *ACTIN2* was used as control. (B—L) GUS staining analysis of *ATRH7pro*:*GUS* transgenic plants. (B) 2-d-old seedling. (C) 5-d-old seedling. (D) 2-week-old plant. (E) to (G) Higher magnification images of (D) showing the surface of the new leaf, the lateral root, and the primary root tip, respectively. (H), (I), and (J) rosette leaf, cauline leaf, and flower of 6-week-old plant. (K) Pollen from (J). Scale bars = 1 mm in (B), (C), (D), (H), (I), and (J); 50 μm in (E), 20 μm in (G), and 10 μm in (F) and (K).

Semi-quantitative RT-PCR was performed to analyze the expression levels of *AtRH7* in different plant tissues. *AtRH7* was expressed ubiquitously in all *Arabidopsis* tissues analyzed (Fig 3A). Transgenic plants expressing *AtRH7pro*:*GUS* were created to investigate tissue-specific expression patterns of *AtRH7*. GUS histochemical assays of the transgenic plants showed that the *AtRH7* promoter drove expression in most tissues of the plants. GUS activity was observed in all tissues of 2- and 5-d-old seedlings (Fig 3B and 3C). In 2-week-old plants, we observed strong GUS staining in rapidly developing tissues including the young rosette leaves, the lateral roots, and the root tips; comparatively weaker activity was also detected in other parts of the plants (Fig 3D–3G). During the reproductive stage, we detected GUS expression in rosette and cauline leaves and floral organs, with strong staining in the stigma, anthers, and pollen (Fig 3H–3K). Thus, our analysis of *AtRH7pro*:*GUS* transgenic plants revealed that expression of *AtRH7* is ubiquitous, with higher levels in rapidly growing tissues.

### *rh7* mutants display growth and developmental alterations resembling those of ribosome-related mutants

To study the biological functions of *AtRH7*, we utilized two T-DNA insertion mutants, *rh7-5* and *rh7-8*, containing insertions in exon 4 and exon 8, respectively (S1A Fig). Disruption of the *AtRH7* expression in these mutants was confirmed by semi-quantitative RT-PCR (S1B Fig).

**Fig 4:**
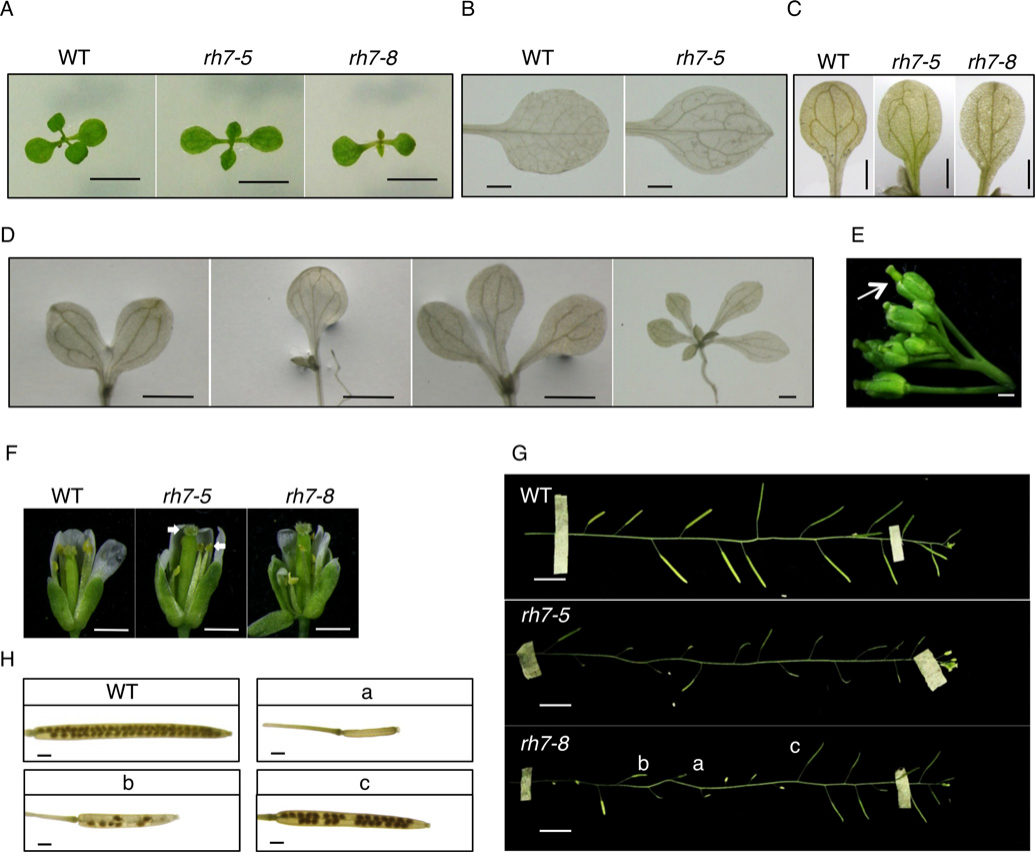
Phenotype analysis of *rh7-5* and *rh7-8* mutants under 22°C. (A) 10-d-old *rh7* mutants displayed narrow pointed first leaves. (B) to (D) Cleared plants of WT and *rh7* mutants. (B) *rh7* mutants show incomplete vascular development with disconnected tertiary and quaternary veins. (C) *rh7* mutants exhibit an aberrant vein pattern in cotyledons, which cannot form closed areoles on the top. (D) Triple, double, fused and quadruple cotyledons of *rh7* mutants. (E) An inflorescence of *rh7-5*; arrow indicates pistil that grew out of buds. (F) Aberrant floral organ of mutants, with stamen filaments shorter than carpel. (G) Siliques from one inflorescence. (H) Cleared siliques of WT and *rh7* mutants, panels (a), (b) and (c) represent different types of siliques from (G). Scale bars = 5mm in (A); 1 mm in (B) to (F) and (H); 1 cm in (G).

Both mutant lines displayed several growth and developmental defects compared with WT. The mutants showed narrow and pointed first true leaves (Fig 4A), and the vascular pattern of these leaves was disturbed, forming more disconnected tertiary and quaternary veins (Fig 4B), a phenotype which resembles those of *rpl4a*, *rpl4d*, *atprmt3*, *apum23* and the nucleolin mutants [37–40]. Cotyledons of the mutants also displayed defects in vein pattern that frequently included opened areoles (Fig 4C). In addition, approximately 1% of the mutants emerged with triple, fused, single, or quadruple cotyledons, which has also been observed in mutants of ribosomal proteins and ribosome biogenesis proteins (Fig 4D) [37,41,42]. Besides the phenotypes observed in shoots, *rh7* mutants also exhibited shorter roots than WT (S2A Fig). Stigmas remain within buds before anthesis in WT, while in *rh7* mutants, the pistil was longer than sepal with a visible stigma (Fig 4E, S2B Fig). In WT, the length of the staminal filament at anthesis should be approximately equal to that of the carpel, so that pollen can be delivered from anther onto stigma. In *rh7* mutants, approximately half of the flowers displayed shorter stamens than that of WT, and this difference might cause the failure of fertilization (Fig 4F). The siliques of the *rh7* mutants developed inconsistently, contained fewer seeds, and exhibited reduced fertility, with some displaying a similar appearance to the WT, but others displaying aborted growth (Fig 4G and 4H). Furthermore, some seeds exhibited slightly shrunken appearance with an abnormal, winkled surface (S2C Fig).

To confirm that the *rh7* phenotypes were caused by the loss-of-function of *AtRH7*, we created a transgenic *rh7-5* mutant that expressed *AtRH7* under the control of its own promoter. A slightly lower level of *AtRH7* expression was observed in the complementation plants than in the WT (S3A Fig). The transgenic plants rescued almost all the phenotypes observed with *rh7-5* (S3B–S3F Fig); however, the root length phenotype was only partially complemented (S3D Fig). Together, these data suggested that AtRH7 plays important roles in plant growth and development.

### Germination and growth defects of *rh7* mutants under cold conditions

**Fig 5:**
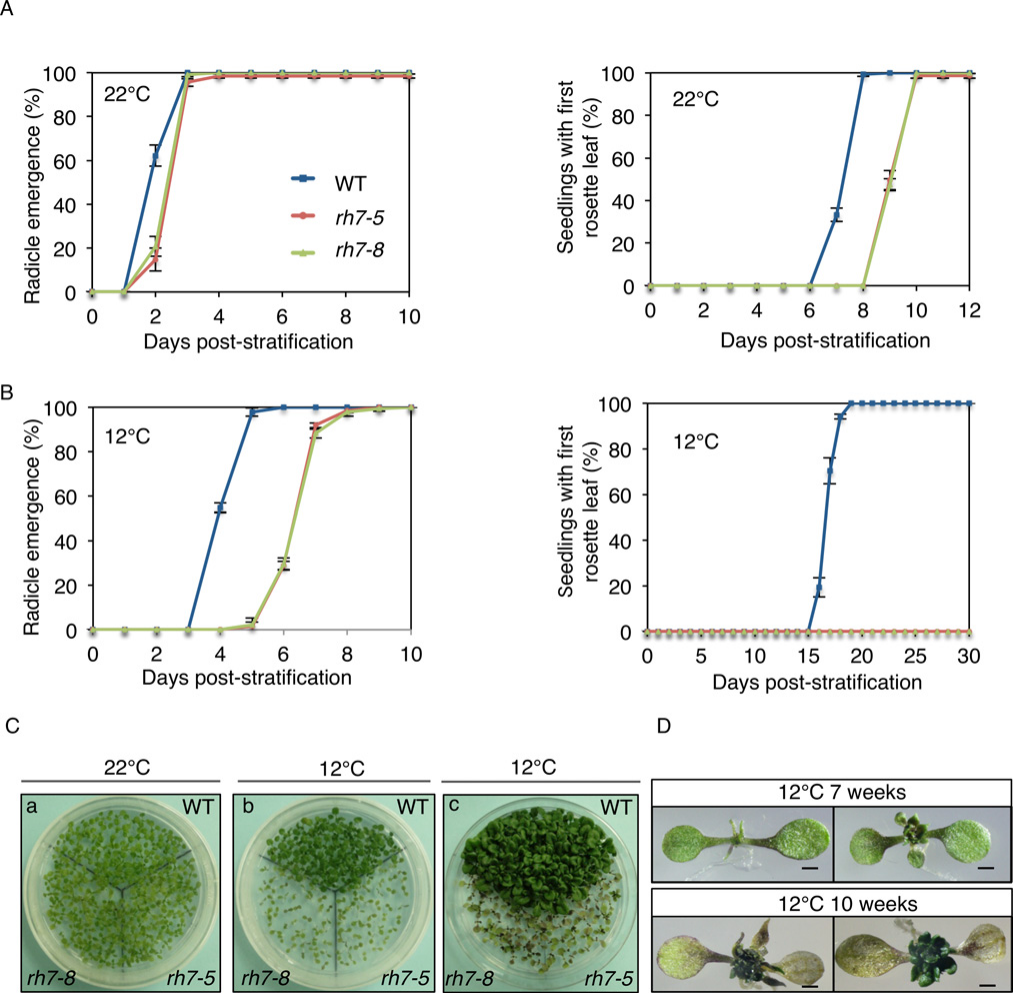
Germination analysis of *rh7* mutants under 22°C and 12°C. (A) Germination of *rh7* mutants at optimal temperature (22°C) compared with WT. (B) Germination of *rh7* mutants and WT at low temperature (12°C). The percentages of germinated seeds in (A) and (B) were counted by the seeds with radicle and first true leaf larger than 1 mm respectively. Each plate had 45 seeds per genotype. (C) Photographs of plates used to generate data in (A) and (B). (a) 2 weeks after germination at 22°C; (b) and (c) 4 and 7 weeks after germination at 12°C. (D) Higher magnification images of mutants germinated at 12°C. Top and bottom panels indicate mutants after germination at 12°C for 7 weeks and 10 weeks, respectively. Note that the first true leaf of *rh7* mutants did not emerge within one month at 12°C, but after prolonged cold treatment, the mutants gradually developed true leaves with an extremely small size and aberrant shape. Scale bar = 1 mm in (D).

To explore whether AtRH7 affects plant growth under cold conditions, we first performed a germination test of WT and *rh7* mutants at normal (22°C) and low (12°C) temperatures. At 22°C, the radicle emergence of *rh7* mutants was slightly delayed as compared with WT. The *rh7* mutants also showed a delay of approximately 2 d in the production of first leaf (Fig 5A). When germination was conducted at 12°C, we found that the delay in radicle emergence in the mutants was extended, as the mutants completed radicle emergence with a 4-d delay relative to WT. Furthermore, *rh7* mutants failed to develop the true leaf within one month, while WT emerged and completed first leaf emergence between the 16th to 20th day (Fig 5B and 5C). To investigate whether AtRH7 regulates seed germination in response to other abiotic stresses, we tested the germination rates of WT and *rh7* mutants under high salt and osmotic conditions. Compared with WT, the germinations of *rh7* mutant seeds under both high salt and osmolality were slightly delayed (S4 Fig), similar to under normal conditions (Fig 5A). Therefore, it appears that AtRH7 may be involved in seed germination specifically under cold stress.

After prolonged growth at 12°C, the mutants eventually developed rosette leaves but they were malformed and did not expand to normal size (Fig 5C and 5D); these types of abnormal leaves were never observed when the mutants were grown at 22°C (Fig 5C). We also compared the growth at 4°C. The WT plants developed three rosette leaves after 6 weeks growth under 4°C, while only the first pair of true leaves emerged in the mutants (S5A Fig). When the growth period was extended to 18 weeks, mutants were much smaller than WT and exhibited yellowish cotyledons (S5B Fig). These results suggested that AtRH7 is required for growth under cold conditions.

**Fig 6:**
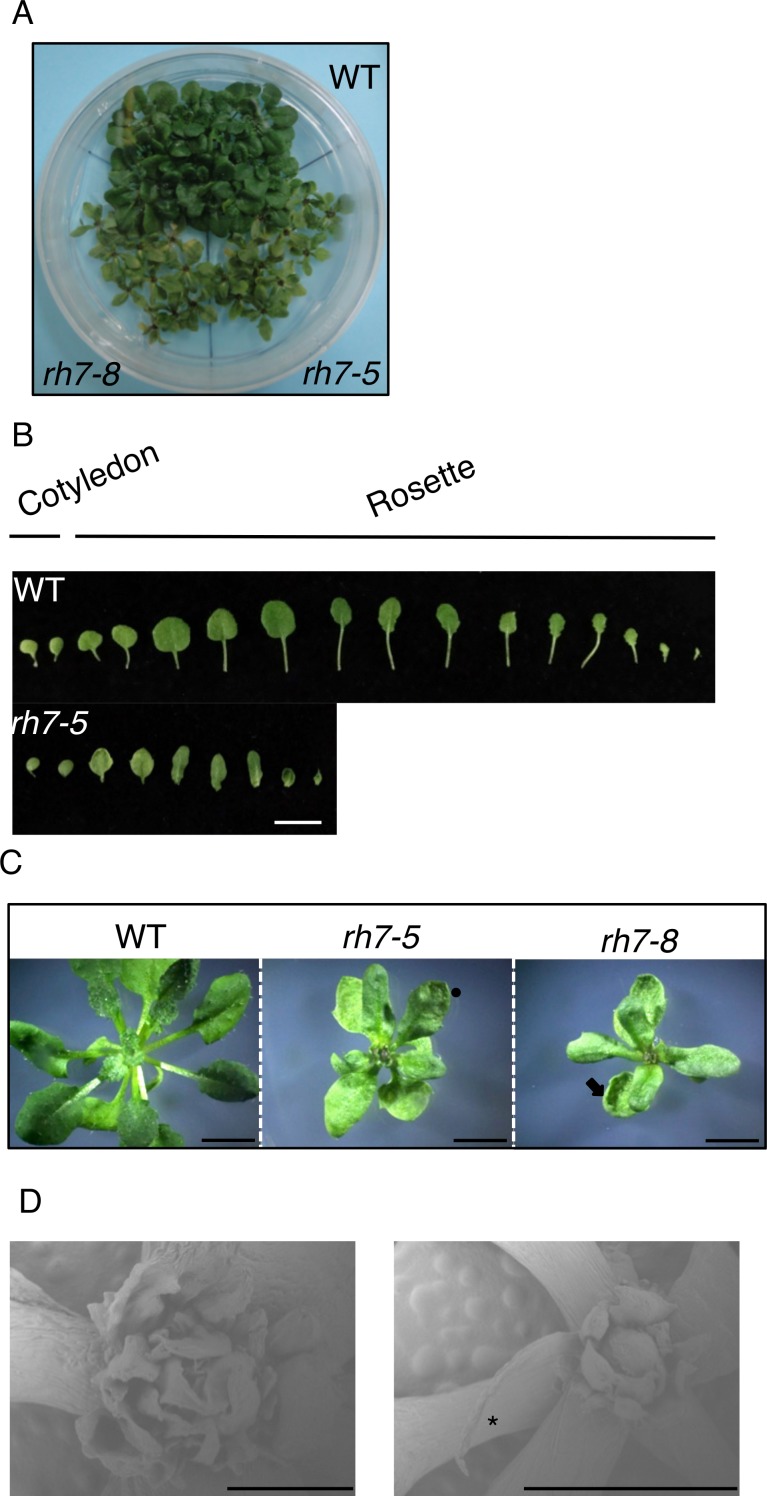
Aberrant leaf phenotype of *rh7* mutants compared with WT under low temperature (12°C). (A) The phenotype of WT and *rh7* mutants germinated at 22°C for 1 week, then transferred to 12°C and incubated for another 5 weeks. (B) Cotyledons and rosette leaves of plants in (A). (C) Typical plants of WT and *rh7* mutants from (A). Circle indicates slightly wrinkly surface; arrow indicates upwardly curling leaf margin. (D) Observation of rosette center of mutants from (A) by scanning electron microscopy. Asterisk indicates a needle-like leaf. Scale bars = 1 cm in (B); 5 mm in (C); 1 mm in (D, left panel) and 2 mm in (D, right panel).

To further characterize leaf morphological alteration at low temperature, 7-d-old seedlings grown at 22°C were transferred to 12°C and further grown up to 5 weeks. The mutant plants exhibited smaller plant stature than the WT or complementation plants (Fig 6A, S3F Fig), and developed fewer total leaves that showed aberrant morphologies such as slightly wrinkled adaxial surfaces, upwardly curling leaves, and, sometimes, short petioles (Fig 6B and 6C). Besides the expanded leaves, SEM analysis revealed that the rosette center of the mutants developed several extremely tiny leaves that did not grow further (Fig 6D). In addition, some leaves displayed needle-like shapes (Fig 6D). When the seedlings germinated at 22°C were transferred to soil and grown at 4°C, the mutant also showed a delay in growth and development, but the phenotype was less severe (S5C Fig).

### AtRH7 affects ribosomal RNA biogenesis in the nucleolus

**Fig 7:**
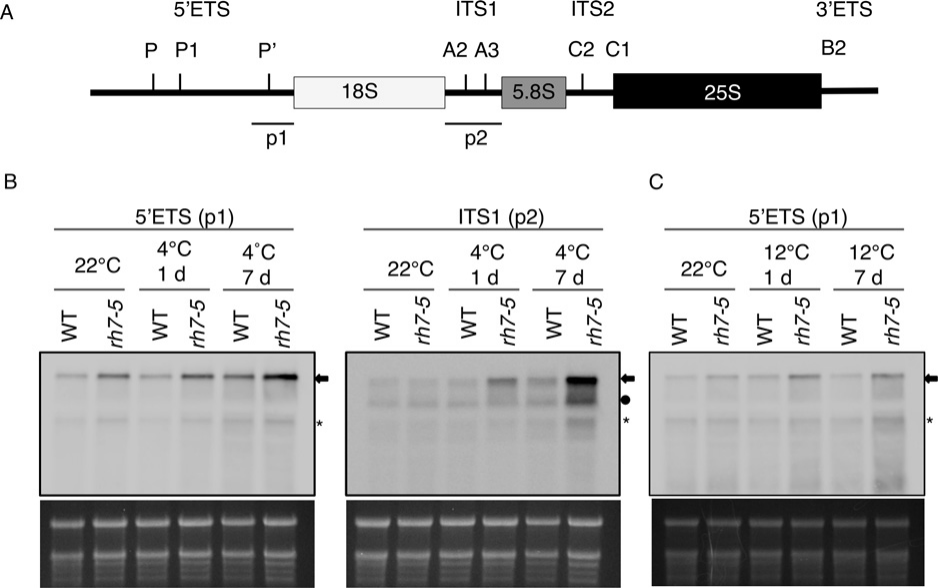
RNA gel blot analysis of pre-rRNA processing. (A) The structure of primary rRNA transcript in *Arabidopsis*. p1 and p2 probes, which specifically bind to 5’ETS and ITS1, respectively, were used for RNA blots. (B), (C) RNA gel blot analysis of 35S pre-rRNA processing. 2-week-old WT and *rh7-5* plants were incubated at 4°C in (B) or 12°C in (C) for an additional 1 d or 7 d. Total RNA isolated from prepared samples was separated in 1.2% agarose, transferred to membranes, and hybridized with the probes indicated in (A). Ethidium bromide-stained rRNA was used as loading control. Arrow indicates 35S pre-rRNA, circle indicates 27SA, and asterisk indicates possible P-A3.

The mutations in *AtRH7* cause several growth and developmental alterations that resemble the phenotypes of mutants defective in ribosomal proteins or factors involved in ribosome biogenesis. This suggested that AtRH7 might be required for rRNA biogenesis. To examine this possibility, we performed RNA blot analysis to determine the accumulation of rRNA precursors in WT and *rh7* mutants. The RNA blots were hybridized with probes that specifically bind to the 5’ external transcribed spacer (5’ETS) or internal transcribed spacer 1 (ITS1) (Fig 7A). We found that the rRNA precursors, most obviously 35S rRNA (indicated by an arrow), accumulated to a higher level in the mutant as compared with WT at 22°C (Fig 7B and 7C, S6 Fig). After 1 and 7 d exposure to low temperatures, the rRNA precursor level was slightly increased in WT. Much higher levels of the 35S, 27SA and possible P-A3 rRNA precursors accumulated in the *rh7* mutant (Fig 7B and 7C, S6 Fig). These data suggested that AtRH7 is involved in rRNA biogenesis, which is affected by low temperatures.

**Fig 8:**
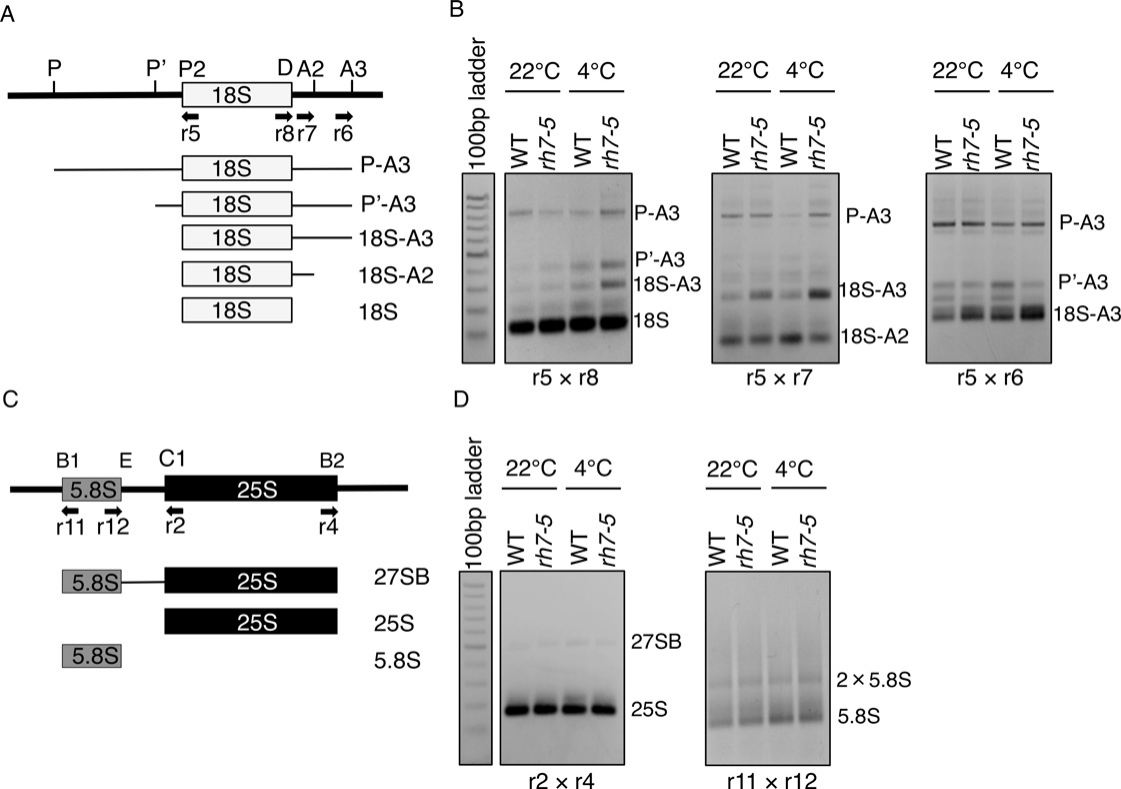
Analysis of 35 pre-rRNA processing by circular RT-PCR. (A) The diagram shows various pre-rRNA processing intermediates amplified by 18S cRT-PCR. (B) Increased accumulation of 18S-A3 intermediates in *rh7-5* mutant. Representative EtBr-stained 1.5% (w/v) gels for 18S rRNA cRT-PCR are shown. The total RNA was isolated from WT and *rh7-5* mutant without and with 7 d 4°C treatment. After RNA circularization and reverse transcribed by 18Sc primer, the pre-18S rRNA intermediates was analyzed by RT-PCR with 5×8, 5×7 and 5×6 primer sets respectively. (C) The diagram of 5.8S rRNA and 25S rRNA intermediates amplified by cRT-PCR. (D) The EtBr-stained gel for 5.8S rRNA and 25S rRNA cRT-PCRs are shown. Various 5.8S rRNA and 25S rRNA intermediates were applied by 11×12 and 2×4 primer sets respectively.

To address the precise step in pre-rRNA processing at which AtRH7 is involved, we performed circular RT-PCR (cRT-PCR) with cDNA synthesized by the primer that is specific to 18S rRNA. As compared to WT, the 18S-A3 intermediate accumulated to a higher level in *rh7* mutant under both normal and cold conditions; however, no consistent results for P-A3 rRNA were observed (Fig 8B, S7 Fig). This suggested the mutation of *AtRH7* affects the 18S rRNA processing in plants. We also analyzed the 25S rRNA and 5.8s rRNA processing by cRT-PCR. As shown in Fig 8D, no clear differences were observed between WT and *rh7* mutant under normal or cold conditions.

Several ribosome biogenesis mutants exhibit resistance to aminoglycoside antibiotics [27,37–39], a phenotype which is caused by aberrations in ribosome function and rRNA processing. We therefore tested whether the *rh7* mutants show resistance to antibiotics. Seedling growth on media containing a range of antibiotics was monitored. As compared with WT, root growth of the *rh7* mutants was less inhibited on the medium containing spectinomycin or streptomycin, indicating that the mutant shows partial resistance against these aminoglycoside antibiotics. No difference in seedling growth was observed on the plates supplemented with gentamicin, hygromycin or the non-amynoglycoside antibiotics, tetracycline and chloramphenicol (S8 Fig). The resistance to spectinomycin and streptomycin in the mutant supported the previous results that AtRH7 is involved in rRNA biogenesis.

## Discussion

RNA helicases are involved in multiple steps of RNA metabolism, such as transcription, splicing, transport, and translation [9]. AtRH7 belongs to a subfamily of DEAD-box RNA helicases that includes human DDX21 and DDX50 together with three other *Arabidopsis* RNA helicases. AtRH7 is most similar to DDX21 and DDX50 and shares a glycine/arginine-rich domain and a GUCT domain in addition to the core DEAD-box region with them. DDX21 participates in several stages of ribosome biogenesis including rRNA transcription and rRNA processing [43]. The other *Arabidopsis* DEAD-box RNA helicases, AtRH3, AtRH9 and AtRH53, in this subfamily localize to chloroplast or mitochondria [21,35]. Accordingly, the localization data suggest that AtRH7 may share a common function with DDX21 in nucleus. Intriguingly, all *E*. *coli* RNA helicases are also included in this subfamily. This subfamily thus appears to represent one of the most conserved DEAD-box RNA helicase class.

AtRH7 was previously identified as an interactor of AtCSP3 [30], which is a cold-inducible RNA chaperone that unwinds double stranded nucleic acids [29]. AtCSP3 can complement the cold-sensitive phenotype of the *E*. *coli* cold shock protein quadruple mutant (*cspA*, *cspB*, *cspE*, *cspG*), indicating functional conservation between plant and bacterial CSD proteins [29]. Similarly, we demonstrated that heterologous expression of *AtRH7* complements the cold-sensitive phenotype of the *csdA* mutant, while another AtCSP3-interacing DEAD-box RNA helicase, AtRH15, does not complement the mutant (Fig 2C). This suggests that AtRH7 and CsdA might share a conserved function under cold conditions. Functional characterization revealed that CsdA is involved in 40S ribosome assembly at low temperature, possibly altering the RNA structure of a 50S precursor [12].

The functional characterization of AtRH7 demonstrated that AtRH7 is a ribosome biogenesis factor that regulates plant growth and development under both optimal and cold conditions (Figs 4, 7 and 8). Recently, Huang et al. [44] reported characterization of *rh7* mutants, and showed several phenotypes that were similar to our observation. They reported that several rRNA precursors accumulated to higher levels in *rh7* mutants than WT. The levels of the precursors further elevated by 4°C treatment in the mutant but not in WT. We also observed accumulation of rRNA precursors in *rh7* mutants (Figs 7 and 8) and the accumulation was elevated in response to cold in both *rh7* and WT, although levels of the accumulation were consistently higher in the mutants. It was reported previously that the level of rRNA precursors were elevated following exposing to low temperature in wild type *Arabidopsis* [45]. Currently, it is unknown how these precursors are accumulated under cold or how AtRH7 can reduce the accumulation. Low temperature can introduce the undesirable secondary structure to RNA [46,47], which may disturb association between pre-rRNA and its processing factors, and cause deficiency in pre-rRNA processing in plants. Therefore, the cold-inducible AtRH7 may unwind the misfolded pre-rRNA, and ensure the proper pre-rRNA processing.

In *Arabidopsis*, deficiency of ribosome biogenesis is associated with resistance to several antibiotics [27,37–39]. Our antibiotics resistance assay showed that *rh7* mutants exhibited increased resistance to spectinomycin in addition to streptomycin that is also reported by Huang et al. [44] (S8 Fig). Spectinomycin and streptomycin target the 16S rRNA of the bacterial ribosome and inhibit protein synthesis [48–50]. Streptomycin can also interact with eukaryotic 18S rRNA [51]. A mutation in yeast 18S rRNA increases its sensitivity to streptomycin [51]. However, it is unknown if spectinomycin can target to eukaryotic 18S rRNA. In *Arabidopsis*, the *rh57* mutants are less sensitive to streptomycin and spectinomycin, and display reduced levels of the 40S ribosomal subunit [27]. Consistent with our cRT-PCR results, the antibiotic resistance of the mutant suggests that AtRH7 might have a function in 40S ribosome biogenesis and assembly.

At normal growth temperatures, the two knockout *rh7* mutant lines displayed several growth and developmental defects including narrow and pointed first leaves, retarded root growth, and disturbed vein patterns. These phenotypes are also observed by Huang et al. [44]. In addition to these phenotypes, we also observed aberrant floral organ development, including a visible anther before anthesis and shorter staminal filaments relative to carpels. Similar phenotypes were also found in several auxin-related mutants, such as *yuc2 yuc6*, *arf1 arf2* and *ap2m* [52–54]. In these mutants, the staminal filaments are shorter than those of WT. Several studies have shown that the morphological alterations caused by mutations in ribosomal proteins or ribosome biogenesis factors contribute to the deficiency of auxin responsiveness. For instance, ribosomal proteins RPL4A, RPL4D, RPL5A, and STV1 (RPL24B) affect translation re-initiation of auxin response factors (ARFs) [55,56]. Nucleolin and *apum23* mutants display altered distribution and reduced expression of the auxin reporter gene DR5-GUS [39,40]. Based on a function of AtRH7 in rRNA biogenesis, it is possible to speculate that the defects in floral organs of *rh7* mutants are a result of the alteration of auxin response caused by aberrant ribosome biogenesis.

AtRH7 has been shown to be essential for growth at 4°C, and *rh7* mutants cannot survive during prolonged 4°C treatment [44]. In this study, we analyzed phenotypes of *rh7* mutants grown at a milder cold temperature (12°C), and found that AtRH7 plays an important role in leaf morphogenesis under cold conditions. At 12°C, the germination and post-germination growth of *rh7* mutants were markedly delayed. When *rh7* seedlings were moved to cold, we observed a variety of leaf abnormalities, including short petiole, curled leaves and needle-like leaves (Fig 6). These data suggested that AtRH7 is required not only for survival of plant in cold but also normal leaf development. Several studies have shown that defects in ribosome biogenesis are associated with a cold-sensitive phenotypes [57–62]. In *Arabidopsis*, REIL1 and REIL2, homologs of the yeast 60S ribosomal maturation factor Reil1p, are required for growth under low temperatures [63]. *Arabidopsis* REIL1 can complement the cold-sensitive phenotype of yeast *Δrei1* [63], and the *Arabidopsis reil1 reil2* double mutant displays delayed germination, and arrested growth and development of the first true leaf under 10°C. The phenotype of the *reil1 reil2* double mutant is in general agreement with the phenotype of *rh7* mutants under 12°C. In addition, the upwardly curled leaves were also observed in *reil2* mutants grown at 10°C for 5 to 6 weeks. Together with the study of REIL1 and REIL2 [63], these data may imply a connection between the regulations of ribosome biogenesis and plant growth, and leave development in the cold.

The interaction of AtRH7 with AtCSP3 was confirmed by *in vitro* and *in vivo* analyses. Intriguingly, interactions between CSD proteins and DEAD-box RNA helicases have been shown in other organisms. In *Bacillus subtilis*, two cold-induced DEAD-box proteins, named CshA and CshB, interact with CSPs to destabilize misfolded RNA and ensure proper initiation of translation [64]. In *Chironomus tentans*, a DEAD-box RNA helicase, hrp84, is associated with a CSD protein, ctYB-1 in nucleus and cytoplasm, and the hrp84-ctYB-1 complex might affect mRNA translation efficiency [65]. Together with AtCSP3-AtRH7, there might be conserved interaction between CSD proteins and DEAD-box RNA helicases in regulation of RNA secondary structures. AtCSP3 positively regulates abiotic stress tolerance through a non-CBF pathway [29,66]. AtRH7 is currently shown to be involved in pre-rRNA processing and chilling tolerance. So far, no common function was found between these proteins. Considering the fact that AtCSP3 and AtRH7 interact within the nucleolus, a plausible function of the AtCSP3-AtRH7 complex is regulation of ribosome biogenesis. This hypothesis is supported by our interactome analysis of AtCSP3 [30]. In the analysis, Gar1, AtNUC-L1 and three ribosomal proteins were identified as interactors of AtCSP3, suggesting that AtCSP3 participates in ribosome biogenesis and rRNA metabolism [30]. The cold-inducible AtCSP3 displays a nucleic acid melting activity in the absence of ATP and functions as an RNA chaperone [29]. AtRH7 has been shown to possess ATP-dependent RNA helicase activity to unwind duplex RNA to single strands [67]. Therefore, AtRH7 and AtCSP3 may cooperatively regulate secondary structures of rRNA within the nucleolus, and ensure the proper pre-rRNA processing in *Arabidopsis*. Although we did not detect any ribosome-related morphological alterations and chilling sensitive phenotype in the *atcsp3* mutant [29], this can be explained by a possible overlapping function with AtCSP1 [68], the other class II CSD protein of *Arabidopsis*, in rRNA processing. Future study should be focused on the function of AtCSP1/3 in chilling tolerance to establish the working model for the AtCSP3-AtRH7 complex in pre-rRNA processing under low temperature.

## Materials and Methods

### Plant materials and growth conditions

*Arabidopsis thaliana* ecotype Columbia was used as wild type (WT) in this study. The T-DNA insertion mutants, *rh7-5* and *rh7-8* (SALK 016729 and SALK 060686, respectively) were obtained from the Arabidopsis Biological Research Center (http://abrc.osu.edu/). The homozygous mutants were analyzed by semi-quantitative RT-PCR using gene-specific primer sets (S2 Table); all primers were synthesized by Hokkaido System Science (Sapporo, Japan). The mutant and WT seeds were surface-sterilized with 75% ethanol for 30 min, and were spread on MS medium containing 2% sucrose and 0.8% agar. The plates were maintained at 4°C for 2 d for stratification, and then moved into a growth chamber set at 22°C (16 h light/ 8 h dark).

### *In vitro* pull-down assay

Production and purification of the GST-AtCSP3 fusion protein was performed as previously described [29]. For preparing the AtRH7-6xHis fusion protein, the entire coding region of *AtRH7* was amplified with AtRH7-pET23(+)F and AtRH7-pET23(+)R primers (S2 Table) and cloned into the BamHI-XhoI site of the vector pET23(+) (Novagen) to yield pET23-AtRH7. *E*.*coli* BL21 (DE3) cells containing pET23-AtRH7 were grown in 50 mL LB with ampicillin at 37°C until an OD_600_ reached 0.5. The recombinant protein was induced by addition of IPTG (0.1mM) and the culture was further growth at 25°C for 4 h. Cells were harvested by centrifugation and resuspended in 5 mL phosphate-buffered saline (PBS). Cells were then disrupted by sonication. Following sonication, crude extract was obtained by centrifugation and subsequently used for pull-down assay. Approximately 1 μg of either GST or GST-AtCSP3 was mixed with the crude extract (100 μL) in PBST (PBS with 1% Triton X-100) to reach a total volume of 200 μL. Then 30 μL glutathione-sepharose was added to the mixture, which was then incubated at 4°C for at least 3 h. After three washes with PBST, the beads were boiled in 2.5×SDS loading buffer and the supernatant was loaded on SDS-PAGE gels. Immunoblotting was performed as previously described [69], and the detection was carried out with anti-6xHis antibodies (MBL, Nagoya, Japan).

### BiFC assay

For BiFC assays, the *AtRH7* ORF was cloned into the EcoRI-BamHI site of pSAT4-cEYFP-N1 [70] to yield cYFP-AtRH7. *AtCSP3* cloned into pSAT4-nEYFP-N1 (nYFP-AtCSP3) was described previously [30]. For subcellular localization analysis, the *AtRH7* ORF was cloned into the SalI site of the pUC18-35S:GFP vector to produce pUC18-35S:AtRH7. Construction of pH7RWG2.0-AtFbr1 was described previously [69]. The subsequent transient expression using onion cells and microscope observation were performed as previously described [30].

### Phylogenetic analysis

Full-length amino acid sequences of DEAD-box proteins from *Arabidopsis*, yeast, human and *E*.*coli* were collected from NCBI (www.ncbi.nlm.nih.gov). We used the NCBI BLASTp (blast.ncbi.nlm.nih.gov/Blast.cgi) conserved domain identification tool to find the core regions of each protein and to exclude the variable N/C termini (blast.ncbi.nlm.nih.gov/Blast.cgi). The amino acid sequences were aligned with Geneious Pro 7.1.8 software (Biomatters, Auckland, New Zealand), and the phylogenetic tree was created with MEGA 6.06 [71] using Neighbor Joining method with a Bootstrap of 1000. The DEAD-box RNA helicases used for phylogenetic tree are list in S1 Table.

### Bacterial complementation

The ORFs of *AtRH7*, *AtRH15*, and *csdA* were cloned into the pINIII expression vector [72] using the NdeI-BamHI site. The plasmids were transformed into *E*. *coli ΔcsdA* mutant cells [33]. The transformants were grown in LB until the OD_600_ reached 0.6. The serially diluted cultures were spotted on LB plates, then the plates were incubated either at 37°C or 17°C, and the growth of cells was observed daily.

### Transgenic expression of *AtRH7pro*:*GUS*

A genomic fragment containing 5’-upstream region of *AtRH7* (2089 bp) was amplified by PCR using the AtRH7*pro* pBI121 Forward and Reverse primers (S2 Table). The PCR product was digested with HindIII and XbaI, and inserted into pre-digested pBI121 vector (Clontech) to make a translational fusion to GUS. The resulting vector was introduced into *Agrobacterium tumefaciens* strain GV3101, and subsequently transformed into *Arabidopsis* plants according to a modified floral-dip method [73,74]. For GUS staining, the tissues from transgenic plants were stained at 37°C overnight in 1 mM 5-bromo-4chloro-3-indolyl-β-D-glucuronide (X-Gluc) solution, then washed several times in 70% ethanol.

### Germination assays

To avoid dormancy variation, WT and *rh7*, seeds were collected from the plants grown under the same conditions. Fully mature seeds were dried and stored at 4°C for at least 1 month. Surface-sterilized WT and *rh7* mutant seeds (45 seeds each) were sown on MS medium. The plates were placed at 4°C for 2 days for stratification, and then were transferred to growth chambers set at 12°C or 22°C under long-day conditions (16 h light/ 8 h dark). The number of seeds with a radicle and first leaf were counted daily. For salt and osmotic stress, the seeds were sown on MS medium containing 130 mM NaCl (for high salt) or 300 mM mannitol (for high osmolality). After stratification, the plates were transferred to 22°C, and the seeds with a radicle were counted daily. For growth under cold, 7-d-old WT and *rh7* mutant plants, which were germinated at 22°C, were transferred to new plates and kept at 12°C or 4°C for the indicated times.

### Electron microscopy

Scanning electron microscopy (SEM) images were obtained using a Hitachi TM-1000 table-top scanning electron microscope (Hitachi High-Technologies Corporation).

### RNA isolation and RNA gel blot analysis

Total RNA was isolated from 12-d-old plants with or without cold treatment using the RNase Plant Mini Kit (Qiagen). RNA blots were prepared as described by Karlson et al. [75] with some modifications. Briefly, 3.5 μg total RNA was separated electrophoretically on 1.2% agarose gels and then blotted onto nylon membranes. Digoxigenin-labeled probes specific to 5ETS and ITS1 (S1 Table) were hybridized to the membrane. The hybridization signals were analyzed using the DIG detection system (Roche Life Science) and results were visualized with a LAS3000 luminoimager (Fuji Photo Film).

### Quantitative real-time PCR (qRT-PCR)

qRT-PCR was performed using an ABI 7500 Real-time PCR systems (Applied Biosystems) with SYBR Premix Ex Taq II (Tli RNnasH Plus) (Takara).The PCR was performed as follow: 95°C for 30 s, 40 cycles of 95°C 5 s, 60°C for 34 s. Three biological replicates for each sample were analyzed, and at least two technical replicates were performed for each biological replicate. Transcripts were normalized to the *ACTIN2*. Primers used in this experiment were listed in S2 Table.

### Circular RT-PCR assay

Circular RT-PCR was performed as previously described [38,39]. Briefly, total RNA was ligated with T4 RNA ligase 1 (New England Biolabs), then the first-strand cDNA was synthesized using the primers specific to 18S rRNA, 5.8S rRNA or 25S rRNA. Various rRNA intermediates were amplified with specific prime sets (S2 Table). Identity of the amplified bands was determined by sequencing.

## Supporting Information

S1 Fig Gene structure and transcript levels of *AtRH7* in two T-DNA insertion lines. (A) Gene structure of *AtRH7* and the positions of the T-DNA insertion in the *rh7* mutants (*rh7-5*/SALK 016729 and *rh7-8*/SALK 060686). The black boxes and lines indicate the exons and untranslated regions, introns respectively. The arrows indicate the primers used for detecting the transcripts by RT-PCR. The triangles represent the T-DNA insertion positions in the SALK lines. (B) RT-PCR analysis of the *AtRH7* transcripts in wild type (WT), *rh7-5* and *rh7-8*. The *ACTIN2* transcript was amplified as control.
(PDF)

S2 Fig Phenotypes of *rh7* mutants. (A) Shorter root length phenotype of *rh7* mutants, 4-d-old plants were incubated vertically at 22°C for a further 1 week. (B) Root length of (A), the results were calculated from three independent experiments, n = 8. The data represent the means ± SD, ** P < 0.01 by *t* test. (C) In some of the *rh7* mutants, the pistil is longer than the sepal. (D) Aberrant surface of *rh7* mutant seeds. Scale bar = 1 cm in (A), 1mm in (C), and 250 μm in (D)
(PDF)

S3 Fig Phenotype analysis of *rh7-5* complementation plants. (A) RT-PCR analysis of *AtRH7* expression level in complementation plants, *ACTIN2* was used as control. (B) Pointed first rosette leaf phenotype of 10-d-old WT, *rh7-5* mutant and complementation plants. (C) Cleared shoots of 10-d-old WT, *rh7-5* and complementation plants. (D) Root length of WT, *rh7-5* and complementation plants incubated vertically. (E) Aberrant floral phenotype. The numerator indicates the number of flowers with normal stamen filament and carpel length, and denominator represents total number of flower observed. (F) Phenotype of WT, *rh7-5* and complementation plants grown at 12°C for 5 weeks. Scale bars = 1 cm in (B) and (F); 1 mm in (C) and (D).
(PDF)

S4 Fig AtRH7 does not affect the germination under high salt and osmotic conditions. The germination of WT and *rh7* mutant seeds after stratification (4°C dark for 2 days) was counted based on the number of seeds with a radicle. Each plate had 45 seeds per genotype.
(PDF)

S5 Fig Growth defects of *rh7* mutants under 4°C. (A), (B) Well-germinated 1-week-old WT and *rh7* mutants were transferred to 4°C, then photographed after 6 weeks (A) and18 weeks (B) following transfer to 4°C. (C) 18-day-old WT and *rh7* mutant were grown in soil at 4°C for 4 months Scale bar = 1 cm.
(PDF)

S6 Fig RNA blot analysis of 35S rRNA and rRNA precursor in mutants and complementation plants. RNA blot analysis of 35S rRNA and rRNA precursor in mutants and complementation plants. RNA was isolated from plants with or without 7-d 4°C treatment. Probes used in this experiment were as described in Fig 7A.
(PDF)

S7 Fig Sequences of rRNA intermediates obtained from cRT-PCR. DNA fragments of cRT-PCR were cloned into pGEM-T Easy vector, and multiple clones were amplified and sequenced.
(PDF)

S8 Fig Antibiotic treatments of WT and *rh7* mutants. WT and *rh7* mutant seeds were directly germinated on plates with or without the indicated antibiotics; then the plates were incubated vertically under long-day conditions in a growth chamber for 2 weeks.
(PDF)

S1 Table DEAD-box RNA helicases used in creating phylogenetic tree. (PDF)

S2 Table Primers used in this work. (PDF)

## References

[1] Fromont-Racine M, Senger B, Saveanu C, Fasiolo F. Ribosome assembly in eukaryotes. Gene. 2003;313: 17–42. 12957375 10.1016/s0378-1119(03)00629-2

[2] Kressler D, Hurt E, Baßler J. Driving ribosome assembly. Biochim Biophys Acta. 2010;1803: 673–683. 10.1016/j.bbamcr.2009.10.009 19879902

[3] Scheer U, Thiry M, Goessens G. Structure, function and assembly of the nucleolus. Trends Cell Biol. 1993;3: 236–241. 14731759 10.1016/0962-8924(93)90123-i

[4] Kressler D, Linder P, de la Cruz J. Protein trans-acting factors involved in ribosome biogenesis in *Saccharomyces cerevisiae*. Mol Cell Biol. 1999;19: 7897–7912. 10567516 10.1128/mcb.19.12.7897 PMC84875

[5] Zemp I, Kutay U. Nuclear export and cytoplasmic maturation of ribosomal subunits. FEBS Lett. 2007;581: 2783–2793. 17509569 10.1016/j.febslet.2007.05.013

[6] Rocak S, Linder P. DEAD-box proteins: the Driving Forces Behind RNA Metabolism. Nat Rev Mol Cell Biol. 2004;5: 232–241. 14991003 10.1038/nrm1335

[7] Cordin O, Banroques J, Tanner NK, Linder P. The DEAD-box protein family of RNA helicases. Gene. 2006;367: 17–37. 16337753 10.1016/j.gene.2005.10.019

[8] de la Cruz J, Kressler D, Linder P. Unwinding RNA in *Saccharomyces cerevisiae*: DEAD-box proteins and related families. Trends Biochem Sci. 1999;24: 192–198. 10322435 10.1016/s0968-0004(99)01376-6

[9] Linder P, Fuller-Pace F V. Looking back on the birth of DEAD-box RNA helicases. Biochim Biophys Acta. 2013;1829: 750–755. 10.1016/j.bbagrm.2013.03.007 23542735

[10] Kaczanowska M, Rydén-Aulin M. Ribosome biogenesis and the translation process in *Escherichia coli*. Microbiol Mol Biol Rev. 2007;71: 477–494. 17804668 10.1128/MMBR.00013-07 PMC2168646

[11] Jain C. The *E*. *coli* RhlE RNA helicase regulates the function of related RNA helicases during ribosome assembly. RNA. 2008;14: 381–389. 18083833 10.1261/rna.800308 PMC2212244

[12] Charollais J, Dreyfus M, Iost I. CsdA, a cold-shock RNA helicase from *Escherichia coli*, is involved in the biogenesis of 50S ribosomal subunit. Nucleic Acids Res. 2004;32: 2751–2759. 15148362 10.1093/nar/gkh603 PMC419605

[13] Rodríguez-Galán O, García-Gómez JJ, de la Cruz J. Yeast and human RNA helicases involved in ribosome biogenesis: current status and perspectives. Biochim Biophys Acta. 2013;1829: 775–790. 10.1016/j.bbagrm.2013.01.007 23357782

[14] Mingam A, Toffano-Nioche C, Brunaud V, Boudet N, Kreis M, Lecharny A. DEAD-box RNA helicases in *Arabidopsis thaliana*: establishing a link between quantitative expression, gene structure and evolution of a family of genes. Plant Biotechnol J. 2004;2: 401–415. 17168887 10.1111/j.1467-7652.2004.00084.x

[15] Gong Z, Dong C-H, Lee H, Zhu J, Xiong L, Gong D, et al A DEAD box RNA helicase is essential for mRNA export and important for development and stress responses in *Arabidopsis*. Plant Cell. 2005;17: 256–267. 15598798 10.1105/tpc.104.027557 PMC544503

[16] Kant P, Kant S, Gordon M, Shaked R, Barak S. STRESS RESPONSE SUPPRESSOR1 and STRESS RESPONSE SUPPRESSOR2, two DEAD-box RNA helicases that attenuate *Arabidopsis* responses to multiple abiotic stresses. Plant Physiol. 2007;145: 814–830. 17556511 10.1104/pp.107.099895 PMC2048787

[17] Kim JS, Kim KA, Oh TR, Park CM, Kang H. Functional characterization of DEAD-box RNA helicases in *Arabidopsis thaliana* under abiotic stress conditions. Plant Cell Physiol. 2008;49: 1563–1571. 10.1093/pcp/pcn125 18725370

[18] Huang T-S, Wei T, Laliberté J-F, Wang A. A host RNA helicase-like protein, AtRH8, interacts with the potyviral genome-linked protein, VPg, associates with the virus accumulation complex, and is essential for infection. Plant Physiol. 2010;152: 255–266. 10.1104/pp.109.147983 19880609 PMC2799361

[19] Guan Q, Wu J, Zhang Y, Jiang C, Liu R, Chai C, et al A DEAD box RNA helicase is critical for pre-mRNA splicing, cold-responsive gene regulation, and cold tolerance in *Arabidopsis*. Plant Cell. 2013;25: 342–356. 10.1105/tpc.112.108340 23371945 PMC3584546

[20] Khan A, Garbelli A, Grossi S, Florentin A, Batelli G, Acuna T, et al The *Arabidopsis* STRESS RESPONSE SUPPRESSOR DEAD-box RNA helicases are nucleolar- and chromocenter-localized proteins that undergo stress-mediated relocalization and are involved in epigenetic gene silencing. Plant J. 2014;79: 28–43. 10.1111/tpj.12533 24724701

[21] Asakura Y, Galarneau E, Watkins KP, Barkan A, van Wijk KJ. Chloroplast RH3 DEAD box RNA helicases in maize and *Arabidopsis* function in splicing of specific group II introns and affect chloroplast ribosome biogenesis. Plant Physiol. 2012;159: 961–974. 10.1104/pp.112.197525 22576849 PMC3387720

[22] Lee K-H, Park J, Williams DS, Xiong Y, Hwang I, Kang B-H. Defective chloroplast development inhibits maintenance of normal levels of abscisic acid in a mutant of the *Arabidopsis* RH3 DEAD-box protein during early post-germination growth. Plant J. 2013;73: 720–732. 10.1111/tpj.12055 23227895

[23] Chi W, He B, Mao J, Li Q, Ma J, Ji D, et al The function of RH22, a DEAD RNA helicase, in the biogenesis of the 50S ribosomal subunits of *Arabidopsis* chloroplasts. Plant Physiol. 2012;158: 693–707. 10.1104/pp.111.186775 22170977 PMC3271760

[24] Nishimura K, Ashida H, Ogawa T, Yokota A. A DEAD box protein is required for formation of a hidden break in *Arabidopsis* chloroplast 23S rRNA. Plant J. 2010;63: 766–777. 10.1111/j.1365-313X.2010.04276.x 20561259

[25] Huang C-K, Huang L-F, Huang J-J, Wu S-J, Yeh C-H, Lu C-A. A DEAD-box protein, AtRH36, is essential for female gametophyte development and is involved in rRNA biogenesis in *Arabidopsis*. Plant Cell Physiol. 2010;51: 694–706. 10.1093/pcp/pcq045 20378763

[26] Liu M, Shi D-Q, Yuan L, Liu J, Yang W-C. SLOW WALKER3, encoding a putative DEAD-box RNA helicase, is essential for female gametogenesis in *Arabidopsis*. J Integr Plant Biol. 2010;52: 817–828. 10.1111/j.1744-7909.2010.00972.x 20738726

[27] Hsu Y-F, Chen Y-C, Hsiao Y-C, Wang B-J, Lin S-Y, Cheng W-H, et al AtRH57, a DEAD-box RNA helicase, is involved in feedback inhibition of glucose-mediated abscisic acid accumulation during seedling development and additively affects pre-ribosomal RNA processing with high glucose. Plant J. 2014;77: 119–135. 10.1111/tpj.12371 24176057 PMC4350433

[28] Nakaminami K, Karlson DT, Imai R. Functional conservation of cold shock domains in bacteria and higher plants. Proc Natl Acad Sci USA. 2006;103: 10122–10127. 16788067 10.1073/pnas.0603168103 PMC1502516

[29] Kim M, Sasaki K, Imai R. Cold Shock Domain Protein 3 Regulates Freezing Tolerance in *Arabidopsis thaliana*. J Biol Chem. 2009;284: 23454–23460. 10.1074/jbc.M109.025791 19556243 PMC2749119

[30] Kim M, Sonoda Y, Sasaki K, Hironori K, Imai R. Interactome analysis reveals versatile functions of *Arabidopsis* COLD SHOCK DOMAIN PROTEIN 3 in RNA processing within the nucleus and cytoplasm. Cell Stress Chaperones. 2013;18: 517–525. 10.1007/s12192-012-0398-3 23334891 PMC3682024

[31] Boudet N, Aubourg S, Toffano-nioche C, Kreis M, Lecharny A. Evolution of Intron / Exon Structure of DEAD Helicase Family Genes in *Arabidopsis*, *Caenorhabditis*, and *Drosophila*. Genome Res. 2001;11: 2101–2114. 11731501 10.1101/gr.200801 PMC311229

[32] Abdelhaleem M, Maltais L, Wain H. The human DDX and DHX gene families of putative RNA helicases. Genomics. 2003;81: 618–622. 12782131 10.1016/s0888-7543(03)00049-1

[33] Awano N, Xu C, Ke H, Inoue K, Inouye M, Phadtare S. Complementation Analysis of the Cold-Sensitive Phenotype of the *Escherichia coli csdA* Deletion Strain. J Bacteriol. 2007;189: 5808–5815. 17557820 10.1128/JB.00655-07 PMC1952031

[34] Henning D, So RB, Jin R, Lau LF, Valdez BC. Silencing of RNA Helicase II/Guα Inhibits Mammalian Ribosomal RNA Production. J Biol Chem. 2003;278: 52307–52314. 14559904 10.1074/jbc.M310846200

[35] Matthes A, Schmidt-Gattung S, Köhler D, Forner J, Wildum S, Raabe M, et al Two DEAD-box proteins may be part of RNA-dependent high-molecular-mass protein complexes in *Arabidopsis* mitochondria. Plant Physiol. 2007;145: 1637–46. 17951454 10.1104/pp.107.108076 PMC2151684

[36] Köhler D, Schmidt-Gattung S, Binder S. The DEAD-box protein PMH2 is required for efficient group II intron splicing in mitochondria of *Arabidopsis thaliana*. Plant Mol Biol. 2010;72: 459–467. 10.1007/s11103-009-9584-9 19960362

[37] Rosado A, Sohn EJ, Drakakaki G, Pan S, Swidergal A, Xiong Y, et al Auxin-mediated ribosomal biogenesis regulates vacuolar trafficking in *Arabidopsis*. Plant Cell. 2010;22: 143–158. 10.1105/tpc.109.068320 20061553 PMC2828701

[38] Hang R, Liu C, Ahmad A, Zhang Y, Lu F, Cao X. *Arabidopsis* protein arginine methyltransferase 3 is required for ribosome biogenesis by affecting precursor ribosomal RNA processing. Proc Natl Acad Sci USA. 2014;111: 16190–16195. 10.1073/pnas.1412697111 25352672 PMC4234621

[39] Abbasi N, Kim HB, Park N-I, Kim H-S, Kim Y-K, Park Y-I, et al APUM23, a nucleolar Puf domain protein, is involved in pre-ribosomal RNA processing and normal growth patterning in *Arabidopsis*. Plant J. 2010;64: 960–976. 10.1111/j.1365-313X.2010.04393.x 21143677

[40] Petricka JJ, Nelson TM. *Arabidopsis* nucleolin affects plant development and patterning. Plant Physiol. 2007;144: 173–86. 17369435 10.1104/pp.106.093575 PMC1913809

[41] Lange H, Sement FM, Gagliardi D. MTR4, a putative RNA helicase and exosome co-factor, is required for proper rRNA biogenesis and development in *Arabidopsis thaliana*. Plant J. 2011;68: 51–63. 10.1111/j.1365-313X.2011.04675.x 21682783

[42] Weis BL, Missbach S, Marzi J, Bohnsack MT, Schleiff E. The 60S associated ribosome biogenesis factor LSG1-2 is required for 40S maturation in *Arabidopsis thaliana*. Plant J. 2014;80: 1043–1056. 10.1111/tpj.12703 25319368

[43] Calo E, Flynn R a., Martin L, Spitale RC, Chang HY, Wysocka J. RNA helicase DDX21 coordinates transcription and ribosomal RNA processing. Nature. 2014;518: 249–253. 10.1038/nature13923 25470060 PMC4827702

[44] Huang C-K, Shen Y-L, Huang L-F, Wu S-J, Yeh C-H, Lu C-A. The DEAD-Box RNA Helicase AtRH7/PRH75 Participates in Pre-rRNA Processing, Plant Development and Cold Tolerance in *Arabidopsis*. Plant Cell Physiol. 2016;57: 175–191. 10.1093/pcp/pcv188 26637537

[45] Shinohara N, Ohbayashi I, Sugiyama M. Involvement of rRNA biosynthesis in the regulation of CUC1 gene expression and pre-meristematic cell mound formation during shoot regeneration. Front Plant Sci. 2014;5: 159–168. 10.3389/fpls.2014.00159 24808900 PMC4009429

[46] Zhu J, Dong C-H, Zhu J-K. Interplay between cold-responsive gene regulation, metabolism and RNA processing during plant cold acclimation. Curr Opin Plant Biol. 2007;10: 290–295. 17468037 10.1016/j.pbi.2007.04.010

[47] Ruelland E, Vaultier MN, Zachowski A, Hurry V. Cold Signalling and Cold Acclimation in Plants Adv in Bot Res. 2009;49: 35–150.

[48] Moazed D, Noller HF. Interaction of antibiotics with functional sites in 16S ribosomal RNA. Nature. 1987;327: 389–394. 2953976 10.1038/327389a0

[49] Honore N, Cloe ST. Streptomycin Resistance in mycobacteria. Antimicrob Agents Chemother. 1994;38: 238–242. 8192450 10.1128/aac.38.2.238 PMC284433

[50] Kehrenberg C, Schwarz S. Mutations in 16S rRNA and ribosomal protein S5 associated with high-level spectinomycin resistance in *Pasteurella multocida*. Antimicrob Agents Chemother. 2007;51: 2244–2246. 17371823 10.1128/AAC.00229-07 PMC1891365

[51] Chernoff YO, Vincent A, Liebman SW. Mutations in eukaryotic 18S ribosomal RNA affect translational fidelity and resistance to aminoglycoside antibiotics. EMBO J. 1994;13: 906–913. 8112304 10.1002/j.1460-2075.1994.tb06334.x PMC394890

[52] Cheng Y, Dai X, Zhao Y. Auxin biosynthesis by the YUCCA flavin monooxygenases controls the formation of floral organs and vascular tissues in *Arabidopsis*. Genes Dev. 2006;20: 1790–1799. 16818609 10.1101/gad.1415106 PMC1522075

[53] Ellis CM, Nagpal P, Young JC, Hagen G, Guilfoyle TJ, Reed JW. AUXIN RESPONSE FACTOR1 and AUXIN RESPONSE FACTOR2 regulate senescence and floral organ abscission in *Arabidopsis thaliana*. Development. 2005;132: 4563–4574. 16176952 10.1242/dev.02012

[54] Kim SY, Xu Z, Song K, Kim DH, Kang H, Reichardt I, et al Adaptor Protein Complex 2 –Mediated Endocytosis Is Crucial for Male Reproductive Organ Development in *Arabidopsis*. Plant Cell. 2013;25: 2970–2985. 10.1105/tpc.113.114264 23975898 PMC3784592

[55] Nishimura T, Wada T, Yamamoto KT, Okada K. The *Arabidopsis* STV1 protein, responsible for translation reinitiation, is required for auxin-mediated gynoecium patterning. Plant Cell. 2005;17: 2940–2953. 16227452 10.1105/tpc.105.036533 PMC1276021

[56] Rosado A, Li R, van de Ven W, Hsu E, Raikhel N V. *Arabidopsis* ribosomal proteins control developmental programs through translational regulation of auxin response factors. Proc Natl Acad Sci USA. 2012;109: 19537–19544. 10.1073/pnas.1214774109 23144218 PMC3511758

[57] Guthrie BYC, Nashimoto H. Structure and Function of *E*.*coli* Ribosomes, VIII. Cold-sensitive Mutants Defective in Ribosome Assembly. Proc Natl Acad Sci USA. 1969;63: 384–391. 4895536 10.1073/pnas.63.2.384 PMC223576

[58] Tai PC, Kessler DP, Ingraham J. Cold-sensitive mutations in *Salmonella typhimurium* which affect ribosome synthesis. J Bacteriol. 1969;97: 1298–1304. 4887510 10.1128/jb.97.3.1298-1304.1969 PMC249847

[59] Waldron C, Roberts CF. Cold-sensitive Mutants in *Aspergillus nidulans* II. Mutantions Affecting Ribosome Production. Mol Gen Genet. 1974;134: 115–132. 4617158 10.1007/BF00268414

[60] Moritz M, Pulaski B A, Woolford JL. Assembly of 60S ribosomal subunits is perturbed in temperature-sensitive yeast mutants defective in ribosomal protein L16. Mol Cell Biol. 1991;11: 5681–5692. 1922070 10.1128/mcb.11.11.5681-5692.1991 PMC361939

[61] Charollais J, Pflieger D, Vinh J, Dreyfus M, Iost I. The DEAD-box RNA helicase SrmB is involved in the assembly of 50S ribosomal subunits in *Escherichia coli*. Mol Microbiol. 2003;48: 1253–1265. 12787353 10.1046/j.1365-2958.2003.03513.x

[62] Woolford JL, Baserga SJ. Ribosome biogenesis in the yeast *Saccharomyces cerevisiae*. Genetics. 2013;195: 643–681. 10.1534/genetics.113.153197 24190922 PMC3813855

[63] Schmidt S, Dethloff F, Beine-Golovchuk O, Kopka J. The REIL1 and REIL2 proteins of *Arabidopsis thaliana* are required for leaf growth in the cold. Plant Physiol. 2013;163: 1623–1639. 10.1104/pp.113.223925 24038679 PMC3850186

[64] Hunger K, Beckering CL, Wiegeshoff F, Graumann PL, Marahiel MA. Cold-Induced Putative DEAD Box RNA Helicases CshA and CshB Are Essential for Cold Adaptation and Interact with Cold Shock Protein B in *Bacillus subtilis*. J Bacteriol. 2006;188: 240–248. 16352840 10.1128/JB.188.1.240-248.2006 PMC1317592

[65] Nashchekin D, Zhao J, Visa N, Daneholt B. A novel Ded1-like RNA helicase interacts with the Y-box protein ctYB-1 in nuclear mRNP particles and in polysomes. J Biol Chem. 2006;281: 14263–14272. 16556597 10.1074/jbc.M600262200

[66] Kim M-H, Sato S, Sasaki K, Saburi W, Matsui H, Imai R. COLD SHOCK DOMAIN PROTEIN 3 is involved in salt and drought stress tolerance in *Arabidopsis*. FEBS Open Bio. 2013;3: 438–442. 10.1016/j.fob.2013.10.003 24251108 PMC3829988

[67] Nayak NR, Putnam A a, Addepalli B, Lowenson JD, Chen T, Jankowsky E, et al An *Arabidopsis* ATP-dependent, DEAD-box RNA helicase loses activity upon IsoAsp formation but is restored by PROTEIN ISOASPARTYL METHYLTRANSFERASE. Plant Cell. 2013;25: 2573–2586. 10.1105/tpc.113.113456 23903319 PMC3753384

[68] Juntawong P, Sorenson R, Bailey-serres J. Cold shock protein 1 chaperones mRNAs during translation in *Arabidopsis thaliana*. Plant J. 2013;74: 1016–1028. 10.1111/tpj.12187 23551487

[69] Sasaki K, Kim M, Imai R. *Arabidopsis* COLD SHOCK DOMAIN PROTEIN2 is a RNA chaperone that is regulated by cold and developmental signals. Biochem Biophys Res Commun. 2007;364: 633–638. 17963727 10.1016/j.bbrc.2007.10.059

[70] Shimizu H, Sato K, Berberich T, Miyazaki A, Ozaki R, Imai R, et al LIP19, a Basic Region Leucine Zipper Protein, is a Fos-like Molecular Switch in the Cold Signaling of Rice Plants. Plant Cell Physiol. 2005;46: 1623–1634. 16051676 10.1093/pcp/pci178

[71] Tamura K, Stecher G, Peterson D, Filipski A, Kumar S. MEGA6: Molecular Evolutionary Genetics Analysis Version 6.0. Mol Biol Evol. 2013;30: 2725–2729. 10.1093/molbev/mst197 24132122 PMC3840312

[72] Xia B, Ke H, Inouye M. Acquirement of cold sensitivity by quadruple deletion of the *cspA* family and its suppression by PNPase S1 domain in *Escherichia coli*. Mol Microbiol. 2001;40: 179–188. 11298285 10.1046/j.1365-2958.2001.02372.x

[73] Clough S.J. and Bent AF. Floral dip: a simplified method for Agro- bacterium-mediated transformation of *Arabidopsis thaliana*. Plant J. 1998;16: 735–743. 10069079 10.1046/j.1365-313x.1998.00343.x

[74] Sasaki K, Kim M, Imai R. *Arabidopsis* COLD SHOCK DOMAIN PROTEIN 2 is a negative regulator of cold acclimation. New Phytol. 2013;198: 95–102. 10.1111/nph.12118 23323758

[75] Karlson D, Nakaminami K, Toyomasu T, Imai R. A cold-regulated nucleic acid-binding protein of winter wheat shares a domain with bacterial cold shock proteins. J Biol Chem. 2002;277: 35248–35256. 12122010 10.1074/jbc.M205774200

