# Supplementary figures and images for: A Cold-Inducible DEAD-Box RNA Helicase from Arabidopsis thaliana Regulates Plant Growth and Development under Low Temperature
Source: PLoS One. 2016 Apr 26;11(4):e0154040. doi: 10.1371/journal.pone.0154040 (PMC4846089; doi:10.1371/journal.pone.0154040)

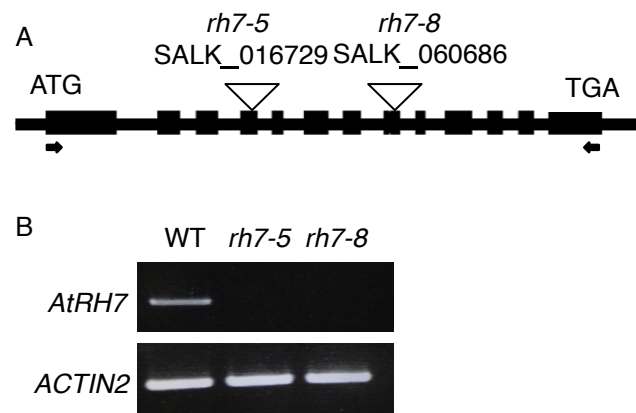

S1 Fig. Gene structure and transcript levels of *AtRH7* in two T-DNA insertion lines.

Supplement: S1 Fig — (A) Gene structure of AtRH7 and the positions of the T-DNA insertion in the rh7 mutants (rh7-5/SALK 016729 and rh7-8/SALK 060686). The black boxes and lines indicate the exons and untranslated regions, introns respectively. The arrows indicate the primers used for detecting the transcripts by RT-PCR. The triangles represent the T-DNA insertion positions in the SALK lines. (B) RT-PCR analysis of the AtRH7 transcripts in wild type (WT), rh7-5 and rh7-8. The ACTIN2 transcript was amplified as control. (PDF) [file pone.0154040.s001.pdf]

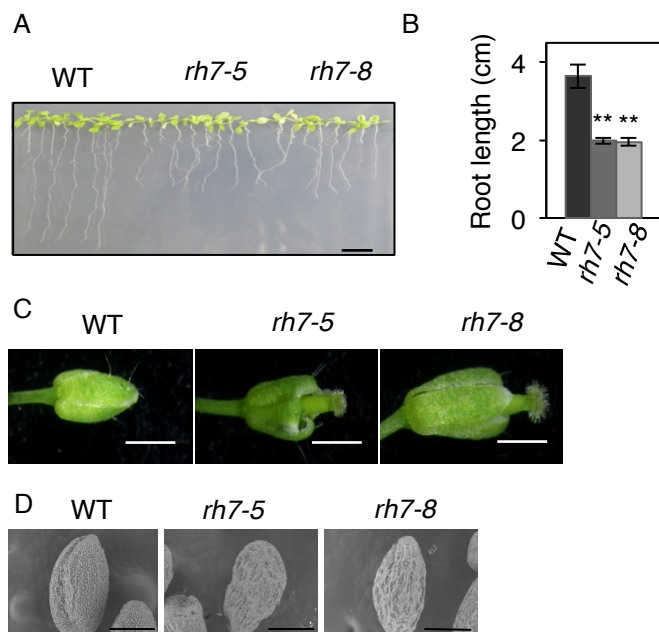

**S2 Fig. Phenotypes of *rh7* mutants.**

Supplement: S2 Fig — (A) Shorter root length phenotype of rh7 mutants, 4-d-old plants were incubated vertically at 22°C for a further 1 week. (B) Root length of (A), the results were calculated from three independent experiments, n = 8. The data represent the means ± SD, ** P < 0.01 by t test. (C) In some of the rh7 mutants, the pistil is longer than the sepal. (D) Aberrant surface of rh7 mutant seeds. Scale bar = 1 cm in (A), 1mm in (C), and 250 μm in (D) (PDF) [file pone.0154040.s002.pdf]

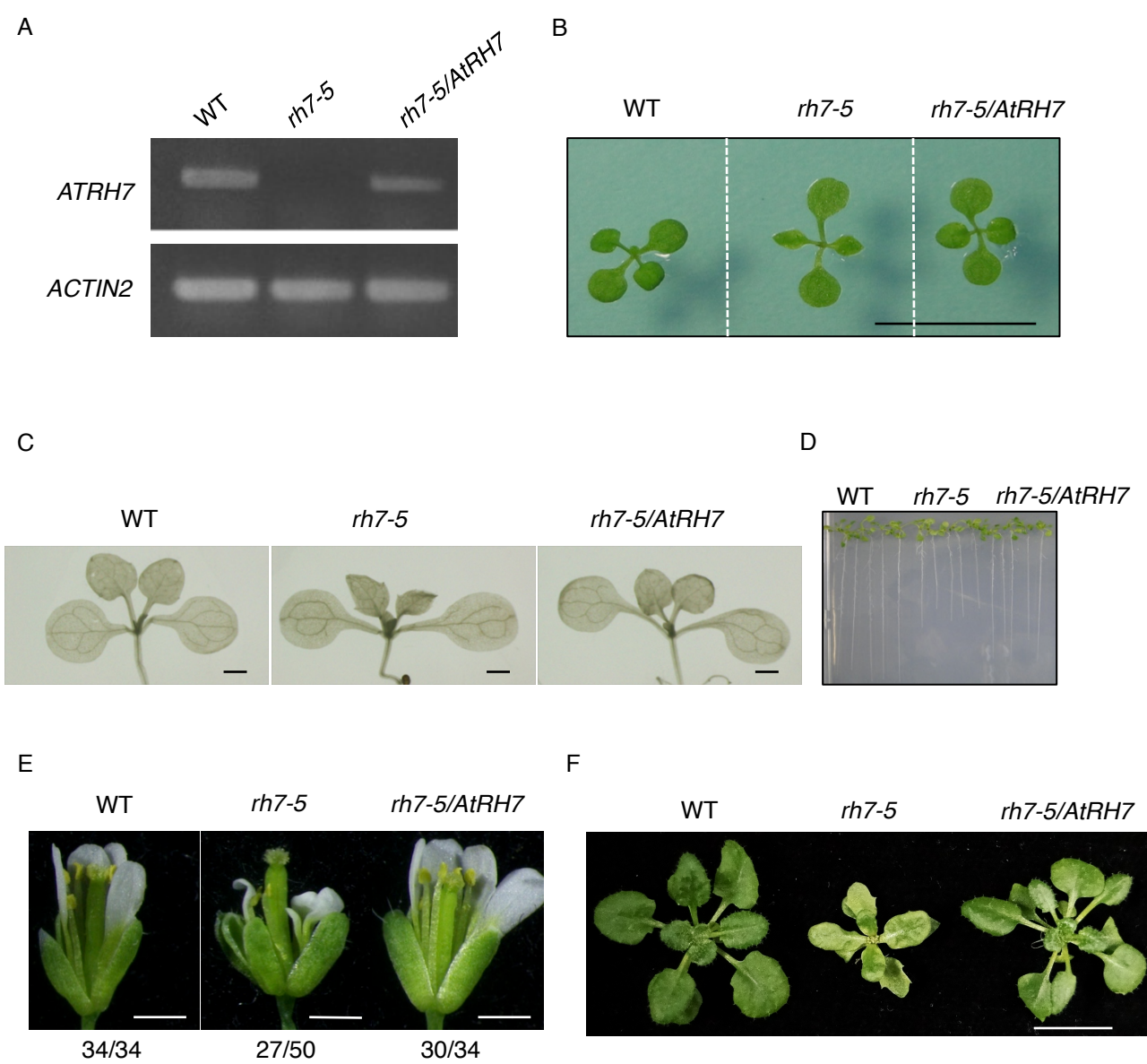

**S3 Fig. Phenotype analysis of *rh7-5* complementation plants.**

Supplement: S3 Fig — (A) RT-PCR analysis of AtRH7 expression level in complementation plants, ACTIN2 was used as control. (B) Pointed first rosette leaf phenotype of 10-d-old WT, rh7-5 mutant and complementation plants. (C) Cleared shoots of 10-d-old WT, rh7-5 and complementation plants. (D) Root length of WT, rh7-5 and complementation plants incubated vertically. (E) Aberrant floral phenotype. The numerator indicates the number of flowers with normal stamen filament and carpel length, and denominator represents total number of flower observed. (F) Phenotype of WT, rh7-5 and complementation plants grown at 12°C for 5 weeks. Scale bars = 1 cm in (B) and (F); 1 mm in (C) and (D). (PDF) [file pone.0154040.s003.pdf]

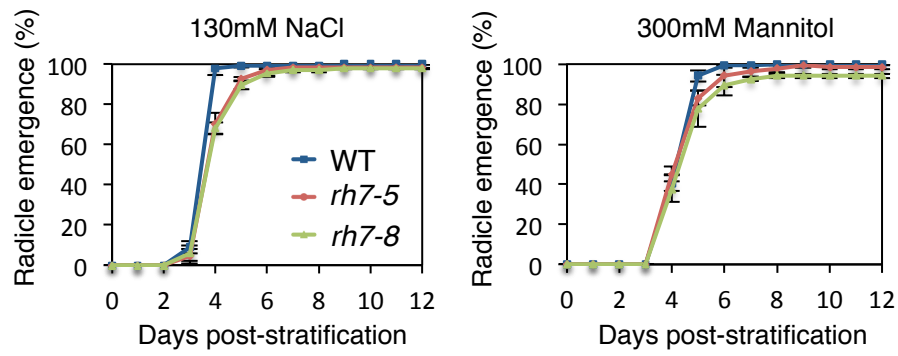

**S4 Fig. AtRH7 does not affect the germination under high salt and osmotic conditions.**

Supplement: S4 Fig — The germination of WT and rh7 mutant seeds after stratification (4°C dark for 2 days) was counted based on the number of seeds with a radicle. Each plate had 45 seeds per genotype. (PDF) [file pone.0154040.s004.pdf]

A

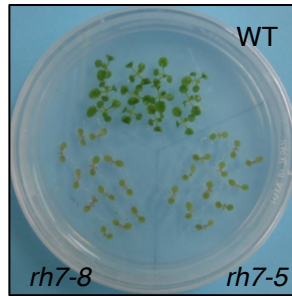

B

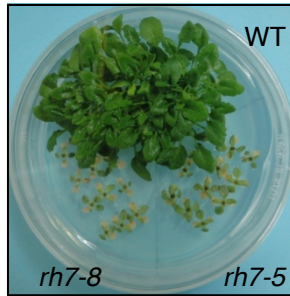

C

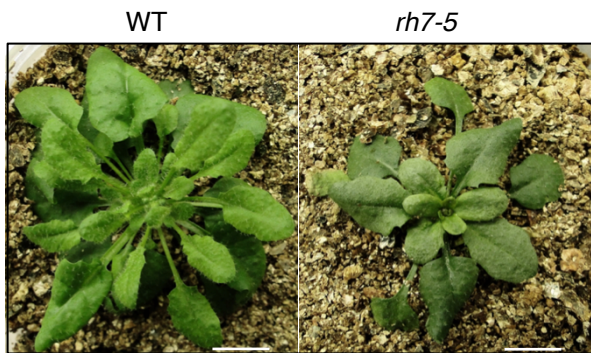

S5 Fig. Growth defects of *rh7* mutants under 4°C.

Supplement: S5 Fig — (A), (B) Well-germinated 1-week-old WT and rh7 mutants were transferred to 4°C, then photographed after 6 weeks (A) and18 weeks (B) following transfer to 4°C. (C) 18-day-old WT and rh7 mutant were grown in soil at 4°C for 4 months Scale bar = 1 cm. (PDF) [file pone.0154040.s005.pdf]

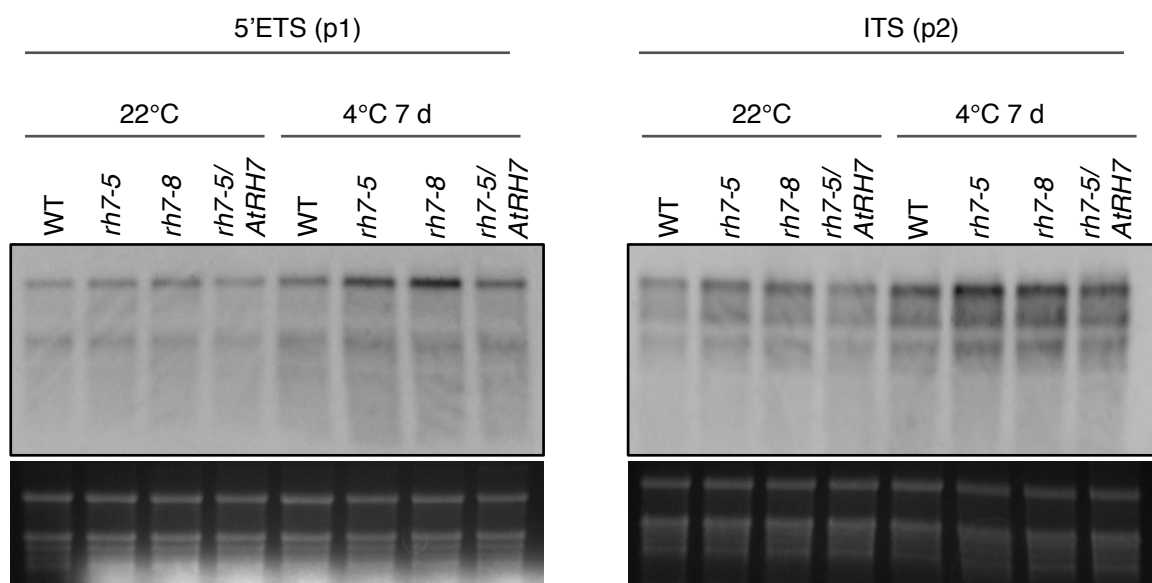

**S6 Fig. RNA blot analysis of 35S rRNA and rRNA precursor in mutants and complementation plants.**

Supplement: S6 Fig — RNA blot analysis of 35S rRNA and rRNA precursor in mutants and complementation plants. RNA was isolated from plants with or without 7-d 4°C treatment. Probes used in this experiment were as described in Fig 7A. (PDF) [file pone.0154040.s006.pdf]

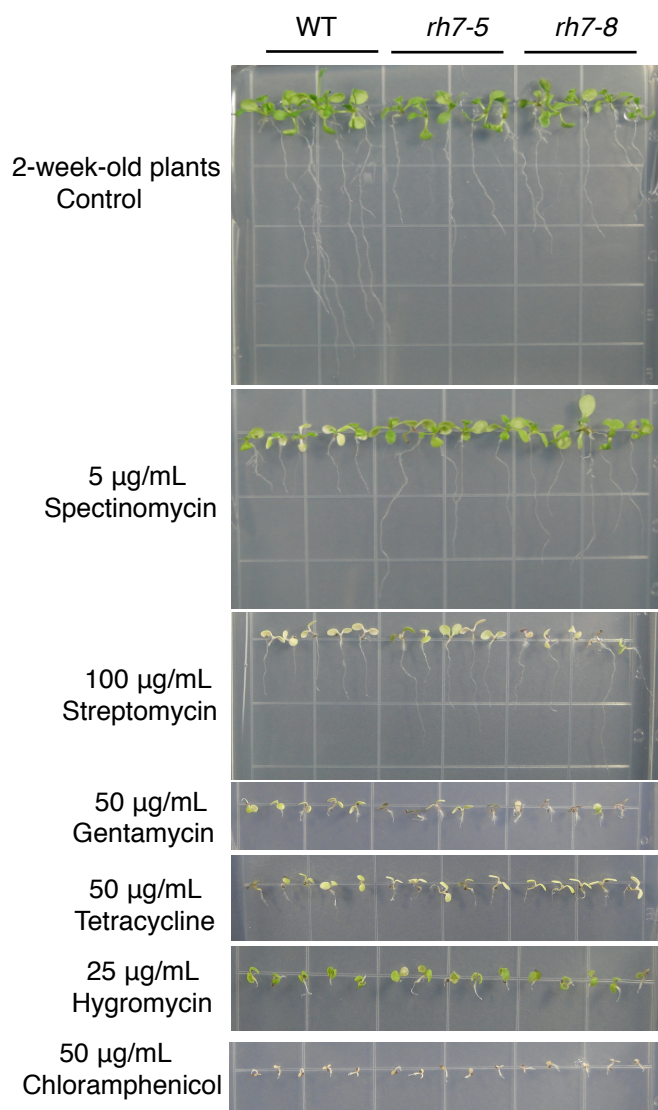

**S8 Fig. Antibiotic treatments of WT and *rh7* mutants.**

Supplement: S8 Fig — WT and rh7 mutant seeds were directly germinated on plates with or without the indicated antibiotics; then the plates were incubated vertically under long-day conditions in a growth chamber for 2 weeks. (PDF) [file pone.0154040.s008.pdf]
