# Supplementary material for: A Cold-Inducible DEAD-Box RNA Helicase from Arabidopsis thaliana Regulates Plant Growth and Development under Low Temperature
Source: PLoS One. 2016 Apr 26;11(4):e0154040. doi: 10.1371/journal.pone.0154040 (PMC4846089; doi:10.1371/journal.pone.0154040)
Supplement: S7 Fig — DNA fragments of cRT-PCR were cloned into pGEM-T Easy vector, and multiple clones were amplified and sequenced. (PDF) [file pone.0154040.s007.pdf]

18S
5' (D site) 3' 5' (P2 site) 3'
·······GGATCATTG-TACCTGGTT······
18S (3' end) 18S (5' end)

18S-A2
5' (A2 site)3' 5' (P2 site) 3'
·······CCAAAACAG-An-TACCTGGTT······
ITS1 18S

18S-A3
5' (A3 site) 3' 5' (P2 site) 3'
·······GTGTCAAGG-An-TACCTGGTT······
ITS1 18S

P'-A3
5' (A3 site) 3' 5' (P' site) 3'
·······CCAAAACAG-An-ATGACGGTC······
ITS1 5'ETS

P-A3
5' (A3 site) 3' 5' (P site) 3'
·······GTGTCAAGG-An-ATCTCGCGC······
ITS1 5'ETS

25S
5' (B2 site) 3' 5' (C1 site) 3'
·······AAGATTCGA-GCGACCCCA······
25S (3'end) 25S (5' end)

27SB
5' (B2 site) 3' 5' (B1 site) 3'
·······AAGATTCGA-An-ACGACTCTC······
25S 5.8S

5.8S
5' (E site) 3' 5' (B1 site) 3'
······GGTGTCACA-An-ACGACTCTC······
5.8S (3'end) 5.8S (5'end)

2 × 5.8S
5' (E site) 3' 5' (B1 site) (E site) 3' 5' (B1 site) 3'
······GGTGTCACA-An-ACGACTCTC······GGTGTCACA-An-ACGACTCTC······
5.8S (3'end) 5.8S 5.8S (5'end)

S7 Fig. Sequences of rRNA intermediates obtained from cRT-PCR.
