# Supplementary material for: A Cold-Inducible DEAD-Box RNA Helicase from Arabidopsis thaliana Regulates Plant Growth and Development under Low Temperature
Source: PLoS One. 2016 Apr 26;11(4):e0154040. doi: 10.1371/journal.pone.0154040 (PMC4846089; doi:10.1371/journal.pone.0154040)
Supplement: S1 Table — (PDF) [file pone.0154040.s009.pdf]

Arabidopsis	ATRH1 ATRH2 ATRH3 ATRH4 ATRH5 ATRH6 ATRH7 ATRH8 ATRH9 ATRH10 ATRH11 ATRH12 ATRH13 ATRH14 ATRH15 ATRH16 ATRH17 ATRH18 ATRH19 ATRH20 ATRH21 ATRH22 ATRH23 ATRH24 ATRH25 ATRH26 ATRH27 ATRH28 ATRH29 ATRH30 ATRH31 ATRH32 ATRH33 ATRH34 ATRH35 ATRH36 ATRH37 ATRH38 ATRH39 ATRH40 ATRH41 ATRH42 ATRH43 ATRH44 ATRH45 ATRH46 ATRH47 ATRH48 ATRH49 ATRH50 ATRH51 ATRH52 ATRH53 ATRH56 ATRH57 ATRH58
Human	DDX1 DDX2A DDX2B DDX3X DDX3Y DDX4 DDX5 DDX6 DDX7 DDX10 DDX17 DDX18 DDX19 DDX20 DDX21 DDX23 DDX24 DDX25 DDX27 DDX28 DDX31 DDX39 DDX41 DDX42 DDX43 DDX46 DDX47 DDX48 DDX49 DDX50 DDX51 DDX52 DDX53 DDX54 DDX55 DDX56
Yeast	Dbp1p Dbp2p Dbp3p Dbp4p Dbp5p Dbp6p Dbp7p Dbp8p Dbp9p Dbp10 Ded1p Dhh1p Drs1p Fal1p Has1p Mak5p Mss116p Prp5p Prp28p Rok1p Rrp3p Spb4p Sub2p Tif1p Tif2p
E.coli	CsdA DbpA RhlB RhlE SrmB

S1 Table. DEAD-box RNA helicases used in creating phylogenetic tree.
