# Supplementary material for: A Cold-Inducible DEAD-Box RNA Helicase from Arabidopsis thaliana Regulates Plant Growth and Development under Low Temperature
Source: PLoS One. 2016 Apr 26;11(4):e0154040. doi: 10.1371/journal.pone.0154040 (PMC4846089; doi:10.1371/journal.pone.0154040)
Supplement: S2 Table — (PDF) [file pone.0154040.s010.pdf]

Supplementary table 2	Primer sets used in this study	
Name	Forward primer 5'-3'	Reverse primer 5'-3'
**RT-PCR**		
AtRH7 full length	ATGCCTTCCCTAATGTTATCTG	ATATCTCTGGCCTCTACCACC
ATRH7 Real Time	GGAGCTGTGTTCGATGTTAAGC	CTTCCAGCACTCTTTTGTGCTC
ACTIN2 Semi RT-PCR	CTTCTTCCGCTCTTTCTTTCCAAG	GAGCTTCTCCTTGATGTCTCTTAC
ACTIN2 Real time	TGTGGATCTCCAAGGCCGAGTA	CCCCAGCTTTTTAAGCCTTTGATC
**BiFC assay**		
AtRH7 pSAT4-cEYFP-N1	GAATTCATGCCTTCCCTAATGTTATCTG	GGATCCAATATCTCTGGCCTCTACCACC
**Subcellular localization**		
GFP-AtRH7	GTCGACATGCCTTCCCTAATGTTATC	GTCGACATATCTCTGGCCTCTACCAC
**Pull-down assay**		
AtRH7 pET23(+)	GGATCCATGCCTTCCCTAATGTTATCTG	CTCGAGTCCATATCTCTGGCCTCT
**Promoter:GUS and complementation**		
AtRH7pro pBI121	AAGCTTTAGAAAAGCACATAACCTACAG	TCTAGATTAGAAGGAAGATAATGGAGAATC
AtRH7 ORF	TCTAGAATGCCTTCCCTAATGTTATC	GAGCTCTCAATATCTCTGGCCTCTAC
**probes for northern blot**		
5ETS	CCTTGCTCGCATTGGTGAATG	CAATTCCCCGCCACATCCTC
ITS1	ACCAAAGATCACCACTCTCGG	AGTTCGCAGCACAGCATCC
**Circular RT-PCR**		
18Sc cRT *	ATGCGTCCCTTCCATAAGTC	
5.8Sc cRT	TCGATGGTTCACGGGATTCTG	
25Sc cRT *	CCGTTACTAAGGGAATCCTTGTTAG	
r5 *	TGCATGGCTTAATCTTTGAGAC	
r6 *	GGTTTCTTAGCCGATTCCTTGC	
r7 *	AGGATCATTGTCGATACCTGTC	
r8 *	AGGAAGGAGAAGTCGTAACAAG	
r2 *	GCAGACGACTTAAATACGCGAC	
r4 *	CTCCGCTTATTGATATGCTTAAAC	
r11	CCAAGTATCGCATTTCGCTACG	
r12	TCTTTGAACGCAAGTTGCGC	
**Complementation of CsdA**		
CsdA pINIII	CATATGGCTGAATTCGAAACC	GGATCCTTACGCATCACCACCGAA
AtRH7 pINIII	CATATGCCTTCCCTAATGTT	GGATCCTCAATATCTCTGGCCT
AtRH15 pINIII	CATATGGGAGACGCTAGAGA	GGATCCTTAAGACGGCATGTAA

* Hang, R., Liu, C., Ahmad, A., Zhang, Y., Lu, F. and Cao, X. (2014) Arabidopsis protein arginine methyltransferase 3 is required for ribosome biogenesis by affecting precursor ribosomal RNA processing. Proc. Natl. Acad. Sci. USA 111:16190–16195
